# NAT10 mediates TLR2 to promote podocyte senescence in adriamycin-induced nephropathy

**DOI:** 10.1038/s41419-025-07515-1

**Published:** 2025-03-19

**Authors:** Mingyang Hu, Linxiao Lv, Yuqi Lei, Min Chen, Sijie Zhou, Zhangsuo Liu

**Affiliations:** 1 Department of Integrated Traditional and Western Nephrology, The First Affiliated Hospital of Zhengzhou University, Zhengzhou, PR China; 2 Research Institute of Nephrology, Zhengzhou University, Zhengzhou, PR China; 3 Henan Province Research Center for Kidney Diseases, Zhengzhou, PR China; 4 Key Laboratory of Precision Diagnosis and Treatment for Chronic Kidney Disease in Henan Province, Zhengzhou, PR China; 5 Tianjian Laboratory of Advanced Biomedical Sciences, Academy of Medical Sciences, Zhengzhou University, Zhengzhou, PR China; 6 Institute of Nephrology, Peking University, Beijing, PR China

**Keywords:** Transcriptomics, Mechanisms of disease

## Abstract

N-acetyltransferase 10 (NAT10) is involved in regulating senescence. However, its role in glomerular diseases remains unclear. Therefore, this study aims to investigate the mechanisms by which NAT10 influences senescence and damage in an adriamycin (ADR)-induced nephropathy model. Senescence (p16 and p21) and DNA damage markers (γ-H2AX (ser139)) were assessed in ADR-induced nephropathy. NAT10 function was demonstrated using Remodelin or small interfering RNA (siRNA) interventions. Transcriptome sequencing was conducted to identify key downstream genes and pathways, while coimmunoprecipitation was performed to evaluate the relationship between NAT10 and toll-like receptor 2 (TLR2) expression. TLR2 overexpression or knockdown further validated its regulatory role in senescence. In ADR-treated mice, the expression levels of P53, P21, P16, γ-H2AX(S139) proteins were elevated, while those of WT-1 and nephrin were reduced. This effect was mitigated by Remodelin and siNAT10 administration. Transcriptome sequencing identified TLR2 as a key downstream gene, and coimmunoprecipitation, along with molecular docking models, confirmed its interaction with NAT10. TLR2 overexpression plasmid or siRNA was employed for recovery experiments. Together, the study findings suggest that NAT10 contributes to podocyte senescence and injury via interaction with TLR2. Further, it demonstrates that NAT10 alleviates ADR-induced podocyte senescence by interacting with TLR2, potentially through a P53-P21-dependent mechanism. Thus NAT10 could serve as a novel therapeutic target for treating podocyte senescence and proteinuric glomerulopathies.

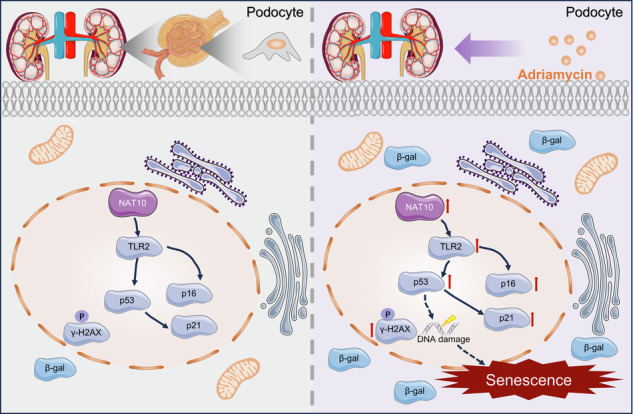

## Introduction

The adriamycin (ADR) nephropathy model is a well-established model for studying focal segmental glomerulosclerosis (FSGS) in clinical settings [1]. FSGS is characterized by diffuse or segmental scarring in most or all glomeruli [2], primarily driven by podocyte damage and loss [3]. Despite extensive research exploring the potential molecular mechanisms underlying FSGS, current clinical treatment strategies still require substantial advancement.

In recent years, concerns have grown over aging-related decline in kidney function. However, the pathogenesis of renal senescence and its related structural and functional changes remain unclear. This uncertainty arises primarily from the difficulty in distinguishing the effects of natural aging from those of toxins or diseases that can damage the kidneys [4]. Recent research indicates that podocyte damage in FSGS mice induces senescence compared with healthy aging mice. Studies have shown that blocking Programmed Cell Death Protein 1(PD-1) signal transduction can reverse the geriatric phenotype of elderly mice and improve proteinuria in FSGS mouse models [5, 6]. This indicates there is a complex interconnection between FSGS and senescence. Besides, Studies have shown that ADR induces podocyte senescence [7]. They found that ADR induces senescence phenotypes and reduce CCAAT Enhancer Binding Protein Alpha (C/EBPα) expression. Furthermore, C/EBPα may regulate podocyte senescence through metabolic mechanisms involving the AMPK/mTOR pathway or inflammatory processes linked to the NLR Family Pyrin Domain Containing 3 (NLRP3) inflammasome. These findings indicate a complex interconnection between ADR-induced FSGS and podocytes senescence. Cellular senescence can occur in physiological and pathological contexts, leading to DNA damage that subsequently triggers the upregulation of the cell cycle inhibitor P21 in a P53-dependent manner or the P16 expression [8].

N-acetyltransferase 10 (NAT10) is an enzyme that catalyzes the acetylation of cytidine (ac4C) in RNA. High NAT10 expression has been observed in several diseases, including Cardiac remodeling and hypertrophy [9], Hepatic steatosis [10], and osteogenic differentiation [11]. Additionally, according to existing research, NAT10 is involved in regulating ferroptosis [12], apoptosis [13], senescence[14], inflammatory responses [15], and nuclear normalization. In recent years, research has shown a significant link between NAT10 and senescence [14, 16–18]. Jackson et al. demonstrated that inhibiting NAT10 improved lifespan in a mouse model of Hutchinson-Gilford Progeria Syndrome (HGPS) [14]. However, the role of NAT10 in podocyte senescence and kidney senescence remains unknown, prompting further investigation into it. Therefore, in this study, we aimed to investigate the specific regulatory mechanisms of NAT10 in ADR-induced podocyte injury using transcriptome sequencing.

In this study, elevated NAT10 expression was observed in an ADR-induced nephropathy model. Results showed that inhibiting NAT10 expression reduces the levels of P53, P21, and the senescent cell marker P16 induced by ADR. Through transcriptome sequencing and experimental validation, toll-like receptor 2 (TLR2) was identified as a key downstream gene regulated by NAT10. Furthermore, our findings showed that NAT10 regulated podocyte senescence and injury by directly interacting with TLR2. The findings could highlight the importance of NAT10 as a potential target in addressing podocyte senescence.

## Results

### Decreased renal function, elevated proteinuria, and glomerular injury and podocyte damage in ADR-injured mice

Urine and blood samples were collected from each mouse before and after ADR or saline injection on days 7 and 14 post-injection, along with body weight measurements. Seven days post-injection, the mice in the ADR10.5 and ADR15 groups exhibited reduced body weight compared to those in the ADR0 group; however, by day 14, the mice in the ADR10.5 group showed some weight recovery (Supplementary Fig. 1A). Analysis of blood albumin levels revealed no significant differences between the ADR10.5 and ADR0 groups. In contrast, the ADR15 group showed significantly lower albumin levels at 7 and 14 days after ADR injection compared to that in the ADR0 group (Supplementary Fig. 1B). Total cholesterol levels in the blood were significantly elevated in the ADR15 group compared to that in the ADR0 group, while no significant change was observed in the ADR10.5 group (Supplementary Fig. 1C). Blood creatinine levels mirrored total cholesterol levels (Supplementary Fig. 1D). Urine protein analysis showed the highest increase in proteinuria in the ADR15 group at 7 days post-injection (Supplementary Fig. 1E). Pathological staining of the kidneys collected on day 14 revealed severe glomerular lesions in the ADR15 group compared to those in the ADR10.5 and ADR0 groups. Western blot (WB) analysis revealed reduced expression of the podocyte markers WT-1 and nephrin in glomeruli exposed to ADR (Supplementary Fig. 1I). Immunohistochemistry (IHC) confirmed a decrease in the WT-1-positive cell count (Supplementary Fig. 1L). These findings indicate significant kidney damage when ADR is administered at a dose of 15 mg/kg.

### NAT10 is overexpressed in glomerular podocytes during ADR-induced cell senescence

**Fig. 1:**
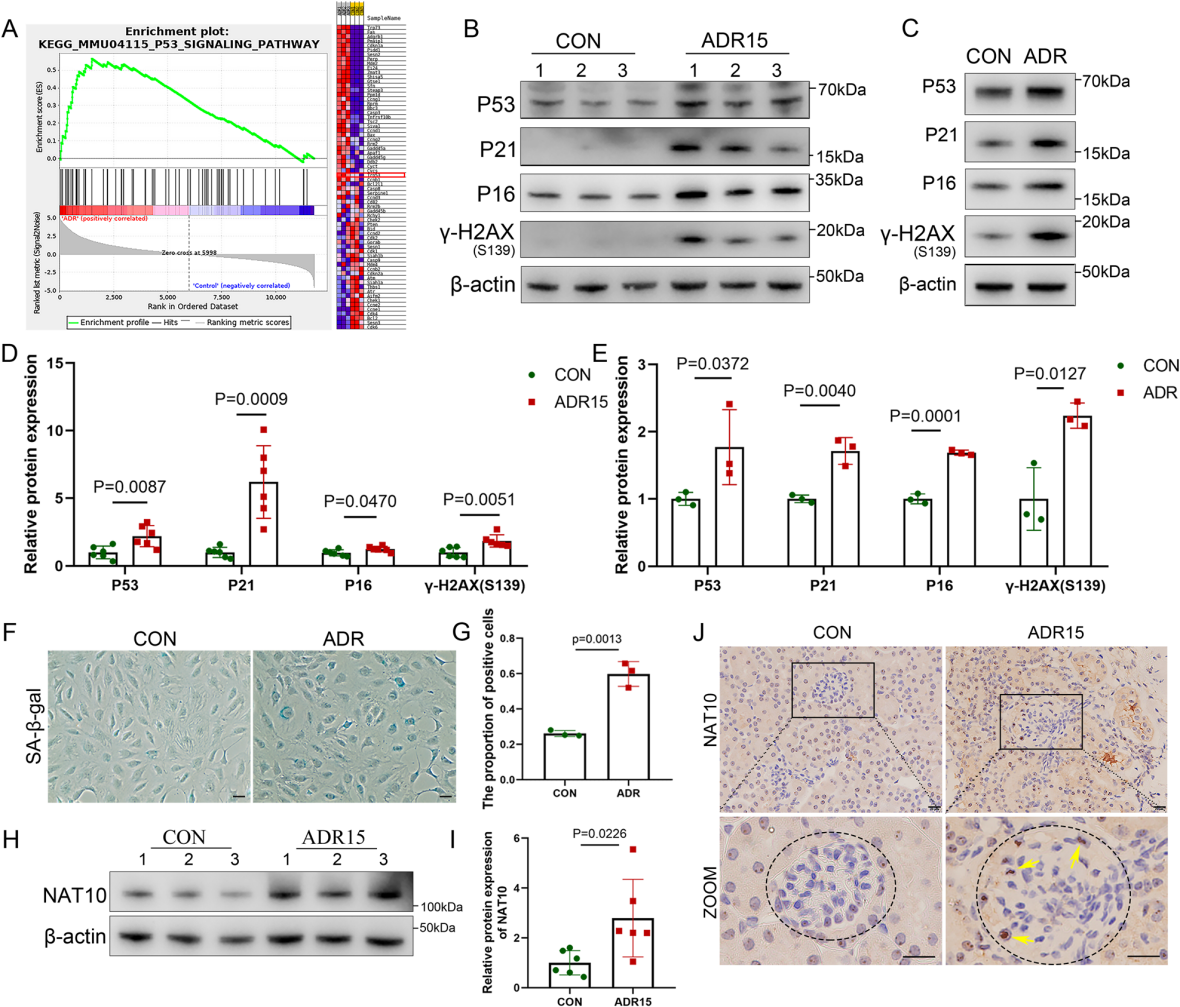
Podocyte senescence induced by adriamycin. **A** Enrichment plots of P53 signaling pathway in podocytes before and after adriamycin stimulation. The stimulation condition of adriamycin was 0.5 μg/mL/12 h. **B** WB was used to detect P53, P21, P16, and γH2AX(S139) expression in the CON group and ADR15 group in kidney tissue (n = 6). **C** WB was used to detect P53, P21, P16, and γH2AX(S139) expression in the CON group and ADR group in podocytes (n = 3). **D** Densitometric analysis of (**B**). The relative intensities of the bands were normalized to the intensities of the respective β-actin signal. **E** Densitometric analysis of (**C**). **F** Representative micrographs showing SA-β-gal staining in podocytes. The scale bars = 20 μm. **G** The quantification of the SA-β-gal-positive cells was determined after three biological replicates. **H** WB was used to detect NAT10 expression in the CON group and ADR15 group in kidney tissue (n = 6). **I** Densitometric analysis of NAT10. **J** Immunohistochemistry was utilized to detect the expression of NAT10. The scale bars = 20 μm.

Transcriptome sequencing revealed differences in the P53 signaling pathway in podocytes before and after ADR stimulation (Fig. 1A). WB was employed to detect changes in the P53 signaling pathway and senescence-related proteins before and after ADR stimulation. The results showed upregulation of P53, P21, P16, and γH2AX(S139) in tissues and cells post-ADR stimulation (Fig. 1B–E). Subsequently, changes in cellular senescence were examined using β-galactosidase (SA-β-gal) staining, indicating an increased proportion of aged podocytes after ADR stimulation (Fig. 1F, G). Further analysis of kidney tissues revealed significantly higher NAT10 expression in the ADR group than in the CON group (Fig. 1H, I). IHC confirmed a marked increase in NAT10 expression within the glomeruli (Fig. 1J). The results of co-immunostaining of NAT10 with podocyte, mesangial cell and endothelial cell marker proteins showed that there was co-staining between NAT10 and the podocyte marker protein WT-1(Supplementary Fig. 2). These findings indicate that ADR induces podocyte senescence and that NAT10 may be involved in its regulation.

### NAT10 inhibition attenuates proteinuria and improves kidney function in ADR nephropathy

To investigate the role of NAT10 in ADR-induced kidney injury, Remodelin as a NAT10 inhibitor was administered via intraperitoneal injection to ADR-treated mice. Biochemical marker levels and renal pathology were assessed, revealing that Remodelin reduced urinary protein, total cholesterol, and blood creatinine levels (Supplementary Fig. 3). Although serum albumin levels in Remodelin treated group were improved compared ADR group, the p-value did not reach statistical significance (Supplementary Fig. 3D). Histological staining of mouse kidneys showed that Remodelin mitigated glomerular damage observed 7 days after ADR injection (Supplementary Fig. 3G). These findings suggest that Remodelin, a small-molecule inhibitor of NAT10, alleviates ADR-induced kidney injury.

### Inhibition of NAT10 protects against podocyte injury with ADR nephropathy

**Fig. 2:**
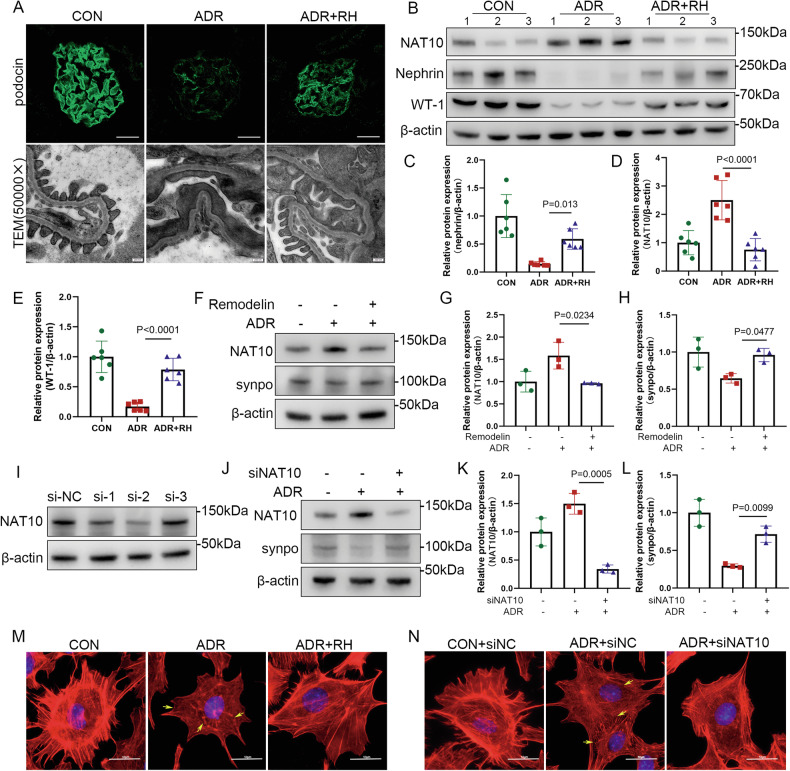
Remodelin alleviates adriamycin-induced Podocytopathy. **A** Representative micrographs show the expression of podocin in different groups with glomeruli. The experiment was performed using frozen slices with a thickness of 5 μm. The scale bars = 20 μm. The electron microscope tissue was obtained from the apical cortex of the mouse kidney. The magnification of the electron microscope is 50,000 times and the scale bars = 200nm. **B** WB was used to detect NAT10, WT-1, and Nephrin expression in the CON group, ADR group, and ADR+RH group in kidney tissue (n = 6). **C** Densitometric analysis of NAT10. **D** Densitometric analysis of WT-1. **E** Densitometric analysis of Nephrin. **F** WB was used to detect NAT10 and Synaptopodin expression in the CON group, ADR group, and ADR+RH group in podocytes (n = 3). **G** Densitometric analysis of NAT10. **H** Densitometric analysis of Synaptopodin. **I** WB was used to detect NAT10 expression in different siRNA treatment conditions. **J** WB was used to detect NAT10 and Synaptopodin expression in different groups (n = 3). **K** Densitometric analysis of NAT10. **L** Densitometric analysis of Synaptopodin. **M**, **N** Immunofluorescence staining of F-ACTIN, red represents F-actin and blue represents DAPI, the scale bars = 50 μm.

To further investigate the therapeutic effects of Remodelin, immunofluorescence staining was performed to assess the expression of podocyte markers podocin. Figure 2A shows that podocin were significantly reduced in this model following ADR stimulation, which was notably alleviated in the treatment group compared to that of the ADR group. Electron microscopy also revealed that Remodelin treatment mitigated ADR-induced podocyte effacement. WB analysis confirmed that Remodelin attenuates podocyte marker protein loss (Fig. 2B–E). Moreover, experiments conducted on cultured podocytes demonstrated that Remodelin reduces synaptopodin loss, a podocyte marker protein (Fig. 2F–H). To elucidate the role of NAT10 in this process, three small interfering RNAs (siRNAs) targeting NAT10 were designed. We found that siRNA-2 had the most significant knockdown effect, which was selected for subsequent cellular intervention (Fig. 2I). WB analysis revealed that siNAT10 partially mitigated ADR-induced loss of synaptopodin (Fig. 2J–L). Podocyte immunofluorescence staining further indicated that the cytoskeletal protein F-actin was fragmented or lost following ADR treatment, an effect attenuated by Remodelin and siNAT10 treatment (Fig. 2M, N). Collectively, these findings suggest that inhibiting NAT10 expression can ameliorate ADR-induced podocyte injury.

### Pharmacologic targeting of NAT10 protects against podocyte senescence in ADR-injured mice

**Fig. 3:**
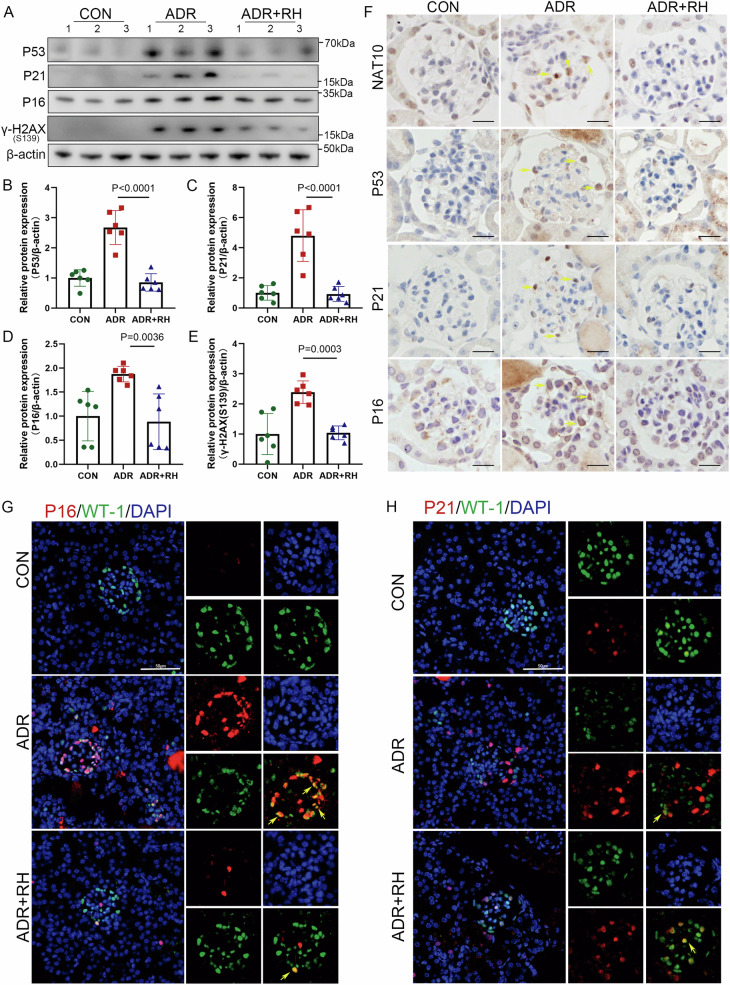
Remodelin protects against podocyte aging in murine models of doxorubicin. **A** WB was used to detect P53, P21, P16, and γH2AX(S139) expression in the CON group, ADR group, and ADR+RH group in kidney tissue (n = 6). **B** Densitometric analysis of P53. The relative intensities of the bands were normalized to the intensities of the respective β-actin signal. **C** Densitometric analysis of P21. **D** Densitometric analysis of P16. **E** Densitometric analysis of γH2AX(S139). **F** Immunohistochemistry was utilized to detect the expression of NAT10, P53, P21 and P16. Yellow arrows point to positive cells. The scale bars = 20 μm. **G** Immunofluorescence staining of P16 and WT-1, red represents P16, green represents WT-1, and blue represents DAPI. The scale bars = 50 μm. **H** Immunofluorescence staining of P21 and WT-1, red represents P21, green represents WT-1, and blue represents DAPI. The scale bars = 50 μm.

Previously, ADR was demonstrated to induce senescence in mouse podocytes. To further investigate the mechanisms underlying this process, changes in senescence-related proteins in the glomeruli were examined following Remodelin treatment. WB analysis revealed that Remodelin attenuated the ADR-induced upregulation of P53, P21, P16, and γH2AX (S139) (Fig. 3A–E). Immunohistochemical staining confirmed that Remodelin suppressed NAT10 expression and reduced the expression levels of P53, P21, and P16 in the glomeruli (Fig. 3F). To demonstrate that these changes occur specifically in podocytes, immunofluorescence staining was performed via co-staining P21 and P16 with the podocyte marker protein WT-1. The results showed colocalization of P16 and P21 with WT-1 (Fig. 3G, H), consistent with the trends observed in WB and IHC analyses. These findings suggest that Remodelin alleviates ADR-induced renal senescence by reducing NAT10 expression in podocytes.

### Pharmacologic inhibition and genetic knockdown of NAT10 protects against podocyte senescence

**Fig. 4:**
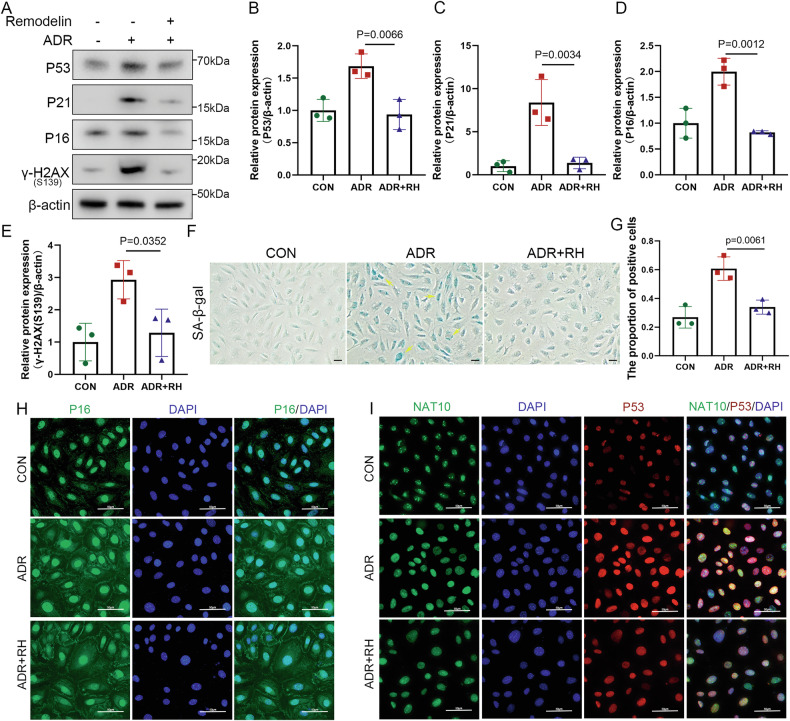
Remodelin attenuates podocyte aging in vitro. **A** WB was used to detect P53, P21, P16, and γH2AX(S139) expression in the CON group, ADR group, and ADR+RH group in podocytes (n = 3). **B** Densitometric analysis of P53. The relative intensities of the bands were normalized to the intensities of the respective β-actin signal. **C** Densitometric analysis of P21. **D** Densitometric analysis of P16. **E** Densitometric analysis of γH2AX(S139). **F** Representative micrographs showing SA-β-gal staining in podocytes in different groups. The scale bars = 20 μm. **G** The quantification of the SA-β-gal-positive cells was determined after three biological replicates. **H** Representative micrographs show the expression of P16 in different groups. Green represents P16 and blue represents DAPI. The scale bars = 50 μm. **I** Representative micrographs show the expression of NAT10 and p53 in different groups. Green represents NAT10, red represents p53, and blue represents DAPI. The scale bars = 50 μm.

The antisenescence effect of Remodelin was further validated in vitro using cultured podocytes. WB analysis revealed that senescence-related proteins—P53, P21, P16, and γH2AX (S139)—increased following ADR stimulation at 0.5 μg/mL for 12 h. Treatment with the NAT10 inhibitor Remodelin at 20 μM for 24 h reduced the elevation of these proteins (Fig. 4A–E). Additionally, SA-β-gal staining of several cell groups demonstrated trends consistent with the WB results (Fig. 4F, G). Furthermore, immunofluorescence assays revealed increased fluorescence intensity of P53 and P16 after ADR treatment, which was mitigated by Remodelin (Fig. 4H, I).

**Fig. 5:**
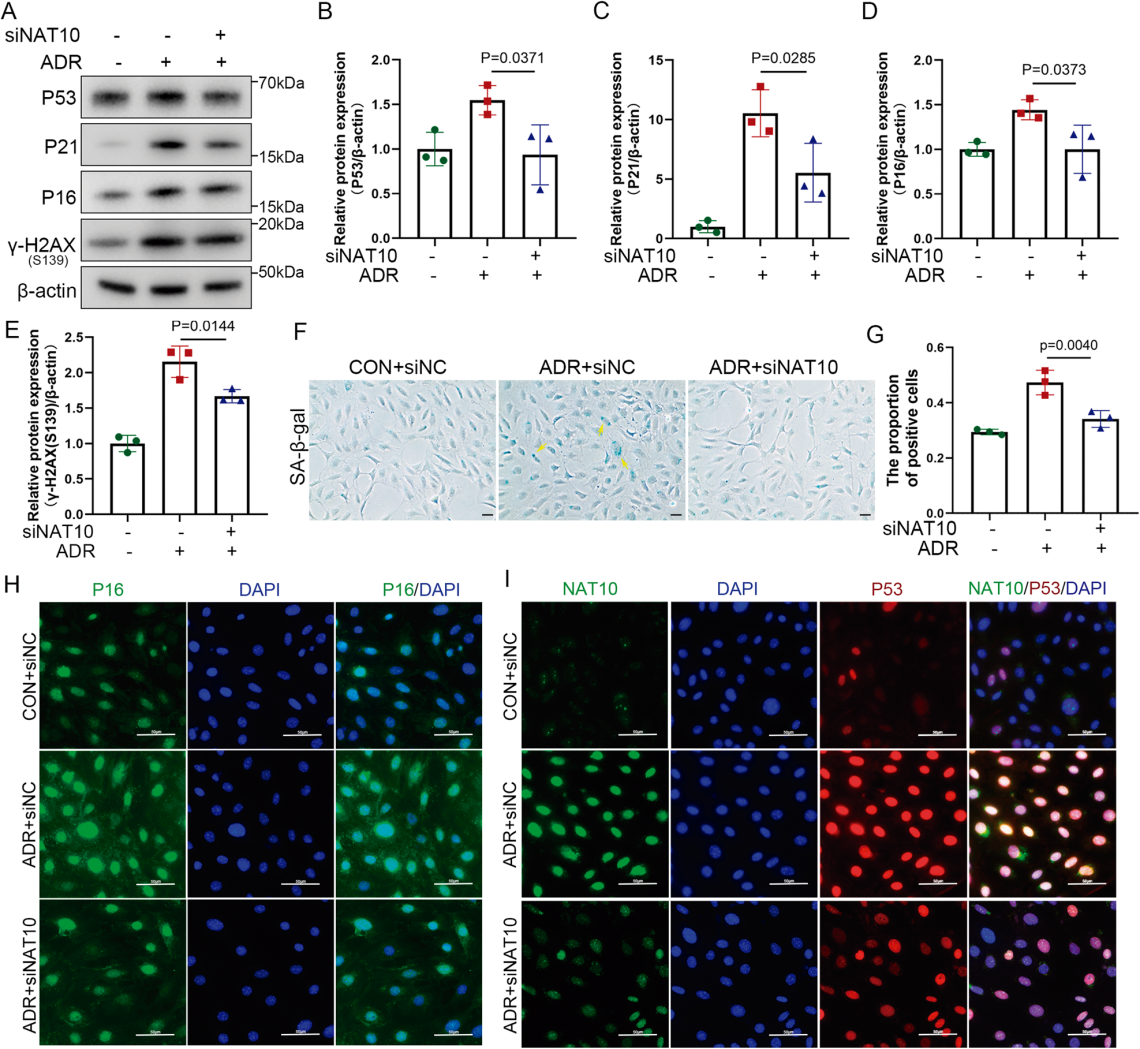
Knockdown of NAT10 alleviates podocyte aging in vitro. **A** WB was used to detect P53, P21, P16, and γH2AX(S139) expression in different groups (n = 3). **B** Densitometric analysis of P53. The relative intensities of the bands were normalized to the intensities of the respective β-actin signal. **C** Densitometric analysis of P21. **D** Densitometric analysis of P16. **E** Densitometric analysis of γH2AX(S139). **F** Representative micrographs showing SA-β-gal staining in podocytes in different groups. The scale bars = 20 μm. **G** The quantification of the SA-β-gal-positive cells was determined after three biological replicates. **H** Representative micrographs show the expression of P16 in different groups. Green represents P16 and blue represents DAPI. The scale bars = 50 μm. **I** Representative micrographs show the expression of NAT10 and p53 in different groups. Green represents NAT10, red represents p53, and blue represents DAPI. The scale bars = 50 μm.

To confirm NAT10 involvement in ADR-induced podocyte senescence, genetic intervention was performed in cultured podocytes, and changes in senescence-related proteins were assessed after ADR treatment or siNAT10 transfection. WB analysis revealed that NAT10 expression knockdown alleviated the ADR-induced increase in P53, P21, P16, and γH2AX (S139) proteins (Fig. 5A–E). Further examination of whether NAT10 knockdown could influence SA-β-gal expression revealed that reducing NAT10 effectively decreased SA-β-gal expression (Fig. 5F, G). Immunofluorescence assays performed on P16 and P53 under siNAT10 conditions revealed that NAT10 knockdown significantly reduced the nuclear expression of P53 and P16 in ADR-stimulated podocytes (Fig. 5H, I). These findings indicate that inhibiting NAT10 can alleviate ADR-induced podocyte senescence.

### TLR2 is involved in NAT10 regulation

**Fig. 6:**
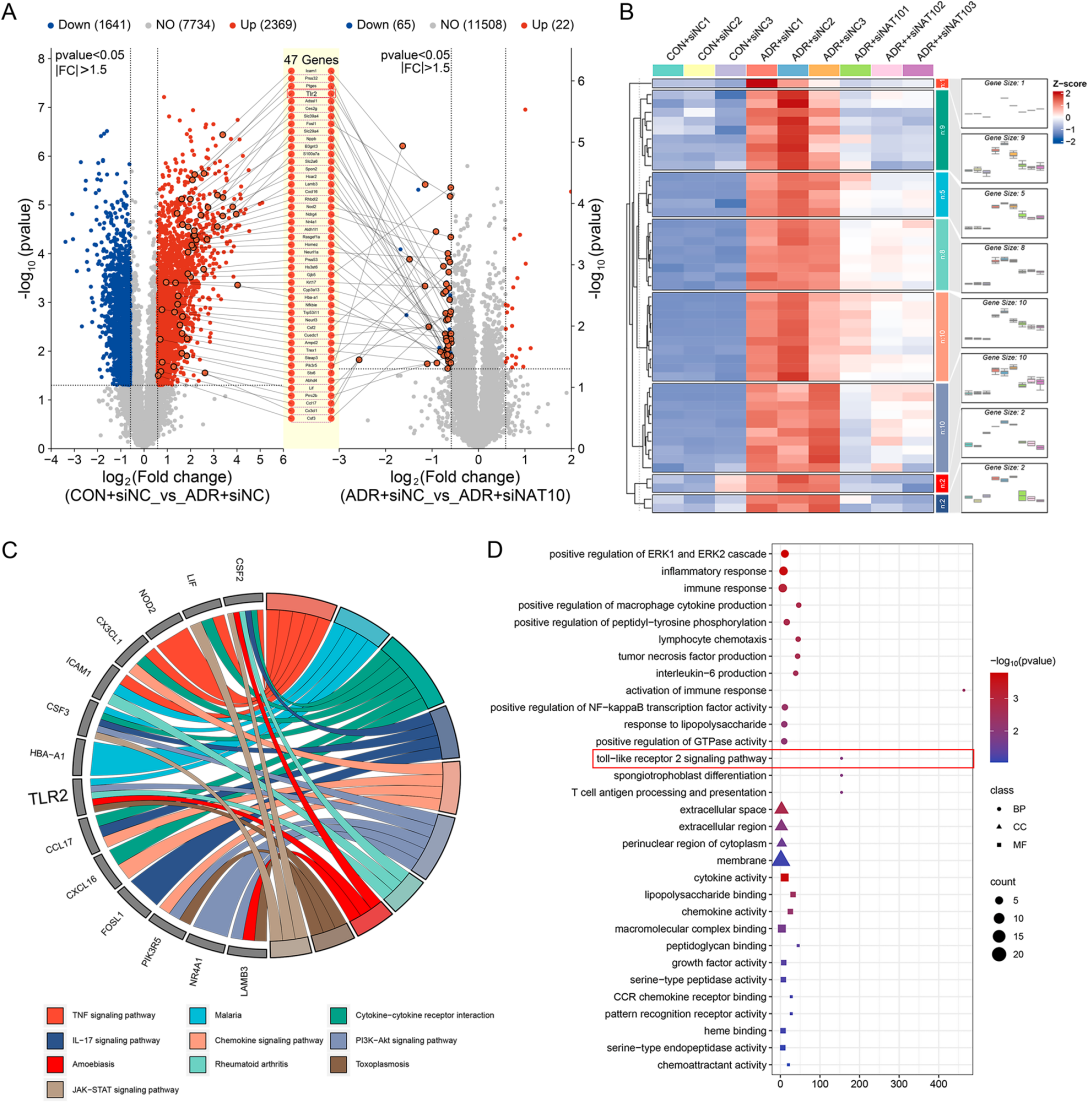
Regulatory network of differentially expressed genes. **A** Volcano plot of gene abundance changes in response to adriamycin and NAT10. A fold change of 1.5 and a P-value of 0.05 were considered critical values. Blue circles represent downregulated genes, and red circles represent upregulated genes. **B** Heatmap of differentially expressed genes. **C** KEGG enrichment of differentially expressed genes. **D** GO enrichment for differentially expressed genes. Circles represent the biological process; triangles represent the cell composition; squares represent the molecular function.

Although NAT10 is involved in podocyte injury and senescence in mouse kidneys, its specific regulatory mechanism remains unclear. Therefore, transcriptome sequencing was performed to explore the specific regulatory mechanisms of NAT10 in ADR-induced podocyte injury. Applying a fold change of 1.5 and a P-value threshold of 0.05, 47 upregulated genes were identified after ADR stimulation but downregulated upon NAT10 inhibition (Fig. 6A). A heatmap was generated for these 47 genes (Fig. 6B). Further analysis of these genes revealed enrichment in several KEGG pathways, including the tumor necrosis factor signaling pathway, interleukin-17 signaling pathway, and PI3K-AKT signaling pathway, with gene TLR2 playing a pivotal role in the regulatory network (Fig. 6C). GO enrichment analysis of the differentially expressed genes revealed enrichment in the TLR2 signaling pathway biological function module (Fig. 6D).

**Fig. 7:**
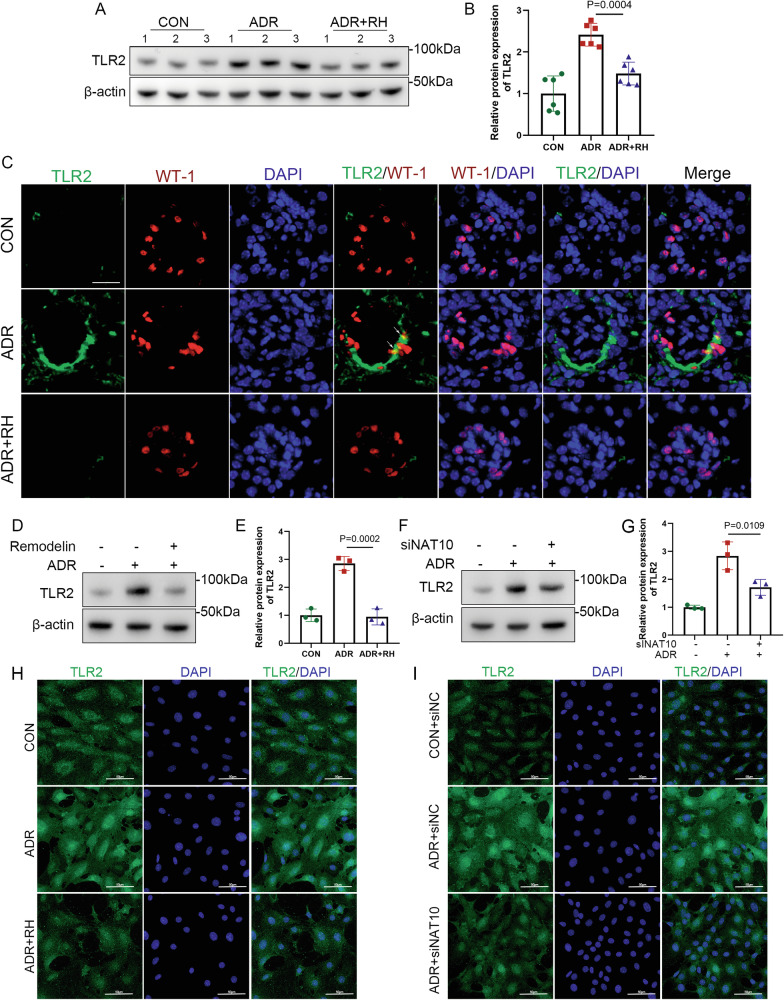
TLR2 is involved in the regulation of NAT10 in adriamycin nephropathy. **A** WB was used to detect TLR2 expression in the CON group, ADR group, and ADR+RH group in kidney tissue (n = 6). **B** Densitometric analysis of TLR2. The relative intensities of the bands were normalized to the intensities of the respective β-actin signal. **C** Representative micrographs show the expression of TLR2 and WT-1 in different groups. Red represents WT-1, green represents TLR2, and blue represents DAPI. The scale bars = 20 μm. **D** WB was used to detect TLR2 expression under adriamycin and Remodelin simulation models. **E** Densitometric analysis of TLR2. **F** WB was used to detect TLR2 expression under adriamycin and siNAT10 simulation models. **G** Densitometric analysis of TLR2. **H** Representative micrographs show the expression of TLR2 under adriamycin and Remodelin simulation models. Green represents TLR2 and blue represents DAPI. The scale bars = 50 μm. **I** Representative micrographs show the expression of TLR2 under adriamycin and siNAT10 simulation models. Green represents TLR2 and blue represents DAPI. The scale bars = 50 μm.

Changes in TLR2 expression were examined in kidney tissues and podocytes. Firstly, the changes in the expression of TLR2 in podocytes under the stimulation of adriamycin were detected by WB. The results showed that the expression of TLR2 were increased in the cytoplasm, plasma membrane, and nucleus after adriamycin induced (Supplementary Fig. 4). Besides, WB showed that TLR2 expression was low in the untreated group (Fig. 7). ADR treatment increased TLR2 expression, whereas NAT10 inhibition reduced TLR2 protein levels. This pattern was consistent in both the kidney tissues and cultured podocytes. Immunofluorescence results were consistent with the WB results (Fig. 7C, H, I). These findings indicate that TLR2 is involved in NAT10-mediated Adrimycin-induced podocyte senescence and injury regulation.

### NAT10-mediated TLR2 to promote ADR-treated podocyte senescence and injury

**Fig. 8:**
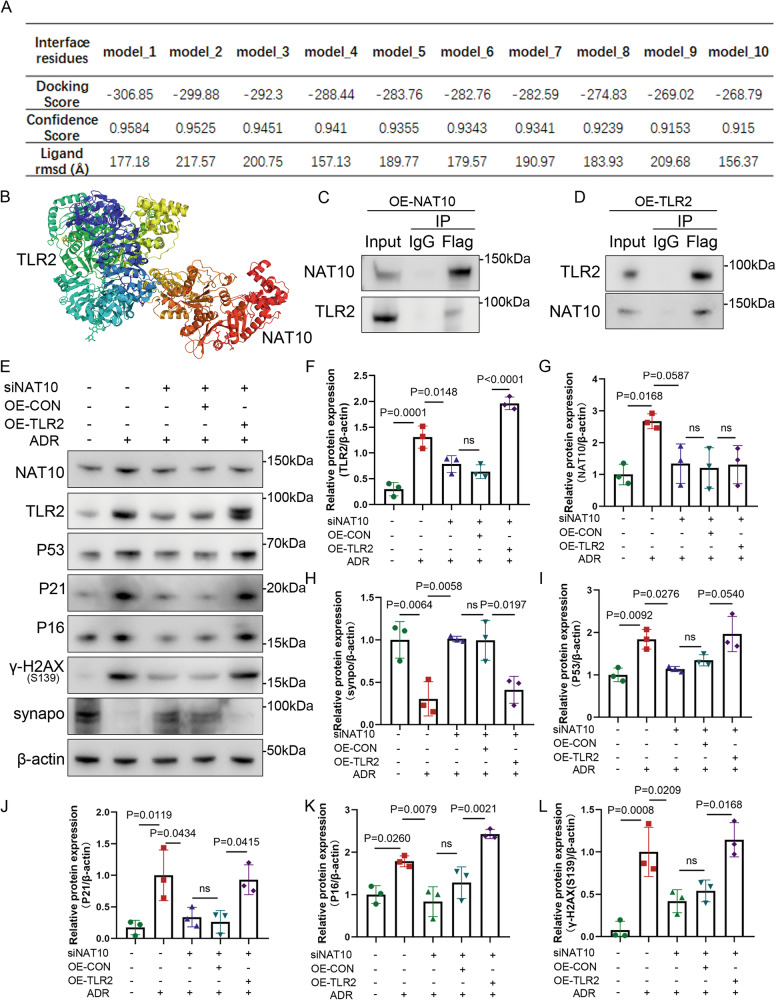
Direct interaction between NAT10 and TLR2. **A** Information on the top ten docking models ranked by docking scores. The docking scores are calculated by the iterative scoring function ITScorePP or ITScorePR. Confidence score = 1.0/[1.0 + e0.02 × (Docking_Score + 150)]. The ligand RMSDs are calculated by comparing the ligands in the docking models with the input or modeled structures. **B** Molecular docking model of NAT10 and TLR2. **C** Immunoprecipitation after transfection with a flag-tagged plasmid overexpressing NAT10. TLR2 and NAT10 were detected by WB. **D** Immunoprecipitation after transfection with a flag-tagged plasmid overexpressing TLR2. TLR2 and NAT10 were detected by WB. **E** WB was used to detect NAT10, TLR2, P53, P21, P16, and γH2AX(S139) expression in different groups (n = 3). The stimulation time of siNAT10 was 36 h, and the transfection time of plasmid overexpressing TLR2 was 48 h. The stimulation condition of doxorubicin was 0.5 μg/mL/12 h. **F** Densitometric analysis of TLR2. The relative intensities of the bands were normalized to the intensities of the respective β-actin signal. **G** Densitometric analysis of NAT10. **H** Densitometric analysis of Synaptopodin. **I** Densitometric analysis of P53. **J** Densitometric analysis of P21. **K** Densitometric analysis of P16. **L** Densitometric analysis of γH2AX(S139).

Studies have shown that NAT10 is a crucial acetyltransferase[19, 20]. To elucidate how NAT10 regulates TLR2, the ac4C modification prediction platform PACES (http://www.rnanut.net/paces/) was used to assess TLR2, which resulted is no acetylated region (Supplementary Material 1). Subsequently, acRIP sequencing of podocytes was performed, but no corresponding peak was detected for TLR2 mRNA (Supplementary Excel). To elucidate the interaction between NAT10 and TLR2, molecular docking was performed. Lower docking scores indicated better binding affinity and the top ten docking models showed favorable scores (Fig. 8A). Figure 8B shows the docking model with the lowest score, suggesting a potential direct interaction between NAT10 and TLR2.

Podocytes were subsequently transfected with a plasmid overexpressing FLAG-tagged NAT10 and enriched using FLAG-tagged magnetic beads (Fig. 8C). These steps indicated that NAT10 could enrich TLR2 protein. To corroborate this finding, podocytes were transfected with a plasmid overexpressing FLAG-tagged TLR2 and similarly enriched (Fig. 8D). The results showed that TLR2 could enrich NAT10 protein, indicating that NAT10 directly interacts with TLR2 to exert its regulatory function.

We demonstrated that NAT10 is involved in ADR-induced podocyte injury and senescence and directly interacts with TLR2. To verify whether NAT10 regulates TLR2 expression, a plasmid overexpressing TLR2 was constructed. The results showed that inhibiting NAT10 mitigated the increase in the expression of P53, P21, P16, and γH2AX (S139) and decrease in that of synaptopodin induced by ADR. TLR2 overexpression diminished NAT10 protective effects (Fig. 8E–L). Additionally, the use of TLR2 siRNA reduced the expression of P53, P21, P16, and γH2AX (S139) upon NAT10 inhibition (Supplementary Fig. 5). In addition, to explore the mechanism by which the NAT10-TLR2 axis regulates DNA damage, we further verified the relationship between TLR2 and the DNA damage regulator P53, and found that there is an interaction between them (Supplementary Fig. 6). Collectively, these findings indicate that NAT10-TLR2 regulate DNA damage by interacting with P53 and then regulate ADR-induced podocyte senescence and injury.

## Discussion

This study highlights the crucial role of NAT10 in podocyte senescence and senescence-induced renal injury. While previous studies suggest a potential link between NAT10 and premature senescence [14, 16, 18], this is the first to clarify its significance in senescence-related renal damage. We found that NAT10 expression increased in ADR-induced podocyte injury and senescence models. Inhibiting NAT10 expression reduced podocyte senescence and damage in vivo, as demonstrated by decreased podocyte senescence marker expression, restored podocin morphology, increased serum albumin levels, lowered blood cholesterol and creatinine levels, and alleviated proteinuria. Studies have shown that TLR2 signaling is crucial for age-related secretory phenotypes and cell cycle arrest, as TLR2 can induce or accelerate senescence [21, 22]. In this study, NAT10 inhibition suppressed TLR2 expression, thus regulating the senescence process. The protective effect of NAT10 inhibition was also confirmed in cultured podocytes.

Cellular senescence is related to various diseases, including pulmonary fibrosis [23], renal diseases [4, 24], obesity-related metabolic syndrome [25], and diabetes [26]. Senescence plays a significant pathogenic role in podocyte injury-related glomerular diseases. In recent years, studies have shown that podocyte senescence exists in glomerular diseases like diabetic nephropathy [27, 28] and hypertensive renal injury [29]. Key features of cellular senescence include stable proliferation arrest mediated by P53 and p16 activation, along with their downstream effectors, p21 and retinoblastoma-1 family proteins. Guido Kroemer and colleagues identified eight molecular changes indicative of aging [30], including lysosomal enlargement (β-galactosidase activation), upregulation of CDK inhibitors (e.g., P16 or P21), and increased DNA damage (increased γ-H2AX). In this study, these three markers were used to assess senescence-related changes. Additionally, Kroeme et al. proposed that cellular senescence is a key response to stress and damage [30]; however, the causal relationship between senescence and damage remains inconclusive. In our opinion, the relationship between senescence and damage is intricate, with each potentially contributing to the other, depending on the state or context.

Studies have confirmed a strong connection between NAT10 and DNA damage. Xie et al. recently reported elevated ac4C RNA levels in cisplatin-treated cells, which induces DNA damage [31]. Remodelin, as a NAT10 inhibitor, decreases DNA damage markers in human lamin A/C-depleted and HGPS-derived patient cells [32]. Additionally, Li et al. found that MORC2 regulates DNA damage [33] and later proved that NAT10 mediates tMORC2 protein acetylation and functions in DNA damage [34]. Researchers have suggested that NAT10 enhances DNA repair in vitro via RNA acetylation-dependent mechanisms [31]. These studies suggest that NAT10 is directly or indirectly involved in DNA damage. In our experiments, inhibiting NAT10 reduced the expression of the DNA damage marker protein γ-H2AX. Previous studies have shown that histone H2AX is rapidly phosphorylated at Ser139 upon double-stranded DNA damage [35, 36]. Histone H2AX at the Ser139 site is also known as gamma H2A.X or γ-H2AX. Therefore, our study confirmed that NAT10 is upstream of DNA damage and repair in ADR-induced in podocytes.

Remodelin was found to repair ADR-induced podocyte damage. As a small-molecule inhibitor, Remodelin has been shown to suppress NAT10 activity in several studies [9, 32, 33]. However, Meier et al. argued that Remodelin is not a specific chemical inhibitor of NAT10-catalyzed RNA acetylation [37]. Recent research indicates that Remodelin suppresses NAT10 expression levels [12, 31, 38], supporting its role as an inhibitor of NAT10 expression. Consistent with these findings, our research revealed that Remodelin decreased NAT10 protein levels in both cultured podocytes and mouse kidney tissues. To further confirm that NAT10 gene changes cause downstream gene alterations, a NAT10 siRNA was used, which yielded results consistent with those obtained from Remodelin treatment.

Current research on NAT10 primarily focuses on its ac4C RNA modifications [39–42]. To investigate whether NAT10 regulates TLR2 through ac4C modification, the TLR2 exon sequence was analyzed using the PACES website, revealing no modifications in this region. Additionally, we performed acRIP sequencing on podocytes cultured in vitro and visualized the immunoprecipitation (IP) relative to the input background reads in the form of peaks. No peaks were detected in the exon region of the TLR2 gene. We speculate that NAT10 may not regulate TLR2 through ac4C modification; however, this speculation needs further validation. Protein-protein interactions are common regulatory mechanisms between proteins. A molecular docking analysis of NAT10 and TLR2 was performed, suggesting a potential interaction between them. Based on this prediction, we overexpressed NAT10 with a FLAG tag and TLR2 with a FLAG tag separately in podocytes, revealing that each protein could enrich the other, confirming an interaction between NAT10 and TLR2 proteins.

Recent studies highlighted a strong link between TLR2 and senescence [21, 22]. A study has shown increased TLR2-positive alveolar cells in aged mice [21]; besides, Hu et al. found that TLR2 absence inhibits liver senescence in hepatocellular carcinoma [22]. Cellular senescence often involves DNA damage, and TLR2 has been shown to activate DNA damage [43], further confirming its association with senescence. In this study, TLR2 expression was increased in the ADR-induced nephropathy model, while Remodelin and NAT10 siRNA treatment reduced TLR2 levels. However, TLR2 overexpression did not increase NAT10 expression, suggesting that NAT10 may regulate senescence by modulating TLR2. However, gene regulatory networks are complex, and this could be just one of many mechanisms that remain under investigation.

Our study has a few limitations. The role of NAT10 was established in an ADR-induced nephropathy model, which simulates the pathological type of focal segmental glomerulosclerosis. However, further investigation is needed to assess the changes in various marker expressions in human samples. Additionally, the study utilized a single time point, which could not fully capture NAT10 function in kidney senescence repair. Therefore, future studies should incorporate multiple time points to provide a more comprehensive understanding of NAT10 function during kidney injury and repair.

In summary, this study revealed the crucial role of NAT10 in regulating DNA damage and senescence-related genes in the ADR-induced nephropathy model. Mechanistic studies showed that NAT10 exerts antisenescence and protective effects by downregulating TLR2 expression. Together, these findings suggest that NAT10 could be a promising therapeutic target for ADR-induced podocyte injury and senescence.

## Materials and method

### Antibodies

The following antibodies were used in this study: anti-β-actin (Zhengneng, #200068-8F10), anti-NAT10 (Abcam, #ab194297), anti-TLR2 (Abcam, #ab209216), anti-P53 (CST, #2524S), anti-P21 (Abcam, #ab109199), anti-P16 (Abcam, #ab189034), anti-gama-H2AX (Ser139) (Abcam, #ab81299), anti-WT-1 (Abcam, #ab89901), anti-nephrin (Abcam, #ab216341), anti-synaptopodin (Proteintech, #21064-1-AP), and anti-podocin (Abcam, #ab50339).

### Animals

Eight-week-old male BALB/c mice were obtained from SPF (Beijing) Biotechnology Co., Ltd (Animal Qualification Certificate Number: SCXK (Beijing) 2024-0001). The mice were housed at 18–24 °C with a 12-h light/dark cycle and acclimated for 1 week before experimentation. The mice were randomly divided into groups. An ADR nephropathy mouse model was established using a single tail vein ADR injection. Two doses (10.5 and 15 mg/kg) were initially selected for the preliminary experiment, with no significant changes observed in the biochemical indices of the mice 14 days after being administered the 10.5 mg/kg injection. Consequently, a dose of 15 mg/kg was selected for subsequent experiments. The mice were randomly divided into three groups: a control group (CON), a 15 mg/kg ADR treatment group (ADR), and an ADR and Remodelin hydrobromide (RH) treatment group (ADR+RH). Each group consisted of six mice. Supplementary Fig. 2A shows a flowchart outlining the mouse modeling process. Blood samples were collected from the mandibular vein, and renal tissues were harvested on day 7 and stored at −80 °C until further use. All animal experiments were approved by the Ethics Committee of the Experimental Animal Center, Zhengzhou University (permission number: ZZU-LAC20230616[05]).

### Cell culture, transfection, and treatment

The mouse podocyte clone-5 cells used in the experiment were purchased from Shanghai Kwaisai Biotechnology Co., Ltd. The cells were cultured in T25 cell culture flasks in a medium containing 1% penicillin, 10% fetal bovine serum, and 90% low-glycemic DMEM (Thermo Fisher Scientific, #11885084). All cells were maintained at a temperature of 37 °C with 5% CO_2_. NAT10 siRNA and overexpression and control TLR2 plasmids were synthesized by HANBIO (Shanghai, China). DNA and siRNA were transfected using Lipofectamine 3000 (Thermo Fisher Scientific, # L3000008) for 48 and 36 h, respectively, following the manufacturer’s instructions. Subsequently, the cells were treated with ADR (MedChemExpress, #HY-B0146) at a concentration of 0.5 μg/mL for 12 h.

### Preparation of cell extracts and WB

The cells were washed twice with pre-cooled phosphate-buffered saline (PBS) (Servicebio, #G4202) solution before sample preparation. Ice-cold radio immunoprecipitation assay lysis buffer (Solarbio, R0010) containing protease and phosphatase inhibitors was then added. The cells were collected, and the cell debris was removed via centrifugation. The resulting supernatant was transferred to a new centrifuge tube. To estimate protein concentration, it was measured using a bicinchoninic acid kit (Solarbio, #PC0020). Cell lysates were denatured by boiling them in a loading buffer at 100 °C.

A 10% separating gel and a 6% concentrated gel was prepared according to the instructions provided in the gel-making kit (YAMEI, #P0012A). Subsequently, 20 µg of protein was loaded per well to the gel, and electrophoresis was performed at 80 V for the concentrated gel and 120 V for the separating gel. Proteins were separated using sodium dodecyl sulfate-polyacrylamide gel electrophoresis (SDS-PAGE) and transferred to a polyvinylidene fluoride membrane (Millipore, #IPVH00010) at 250 mA voltage for 90 min. The membrane was blocked with 5% skim milk and washed thrice with TBST for 5 min each. The primary antibody (NCM Biotech, #WB500D) was added at a 1:1000 ratio and incubated overnight at 4 °C. The following day, after three washes with TBST, the membrane was incubated with the secondary antibody at room temperature for 1 h. Finally, the protein bands were visualized using enhanced chemiluminescence (Tanon, #180-506) on a Fusion FX SPECTRA multifunctional imaging system. The uncropped WB bands and their three biological replicate bands in the results are in Supplementary Material 1.

### Co-immunoprecipitation

The FLAG-tagged immunoprecipitation kit (China, Beyotime, #P2181S) was purchased from Beyotime Corporation. According to the manufacturer’s instructions, processed cells were lysed with 600 μL of lysis buffer (mixed with protease inhibitor at a 100:1 ratio) per T75 flask. The protein samples from each group were divided into three parts: 60 μL for the input group and 250 μL each for the IgG and IP groups. The IgG and IP groups were incubated overnight at 4 °C on a shaker with their respective magnetic beads. The following day, the beads were washed three times, separated, and the supernatants were discarded. Each IgG and IP sample was mixed with 50 μL of 1× SDS-PAGE sample loading buffer, while the input group was mixed with 15 μL of 5× SDS-PAGE sample loading buffer. All samples were denatured at 95 °C for 5 min, and the supernatants were separated using a magnetic rack. Subsequently, WB was performed for sample analysis.

### IHC

IHC experiments were performed on 5 μm thick kidney tissue sections as follows: (1) Baking: Tissue sections were placed in a 60 °C oven for 1 h. (2) Deparaffinization: The slides were deparaffinized with xylene, followed by immersion in graded ethanol concentrations for 5 min each, and then washed three times in PBS for 5 min per wash. (3) Antigen retrieval: Tris-EDTA antigen retrieval buffer at pH 9 was used with microwave treatment for 1 min at high power, 3 min at medium power, and 10 min at low power, followed by natural cooling for subsequent steps. (4) Endogenous peroxidase blocking: Tissues were incubated in 3% H_2_O_2_ for 20 min, then washed three times with PBS for 5 min each. (5) Permeabilization: Samples were treated with 0.1% Triton X-100. (6) Blocking: Samples were blocked with goat serum. (7) Primary antibody incubation: Different groups of slides were incubated overnight at 4 °C with the corresponding primary antibodies. (8) Secondary antibody incubation: Before secondary antibody incubation, slides were treated with a reaction enhancer for 20 min at 37 °C, followed by three 5 min washes with PBS. Secondary antibodies were applied according to the species origin of the primary antibodies. (9) DAB staining: The DAB staining time was controlled according to the manufacturer’s specifications. (10) Hematoxylin counterstaining: The cell nuclei were stained with hematoxylin. (11) Dehydration: Slides were dehydrated in graded ethanol concentrations and cleared in xylene. (12) Mounting: Slides were mounted with neutral gum, and images were captured under a microscope.

### Immunofluorescence

Immunofluorescence was performed on frozen sections of Optimal cutting temperature compound-embedded kidney tissue cut to 5 μm using a frozen microtome. Sections were fixed with pre-cooled methanol for 10 min. Bovine serum albumin (5%) was incubated in a 37 °C-temperature box for 30 min and incubated with the corresponding primary antibody overnight at 4 °C. The following day, the primary antibody was removed, and the sections were soaked thrice in PBST for 5 min each. The sections were then incubated with a secondary antibody at room temperature in the dark for 1 h, followed by three additional washes with PBST for 5 min each. Nuclei were stained with DAPI for 5 min, soaked, and washed three times with PBST, and the slices were dried and sealed.

### Transcriptome sequencing

Total RNA samples were extracted and assessed for quality using agarose gel electrophoresis, followed by quantification with a nanodrop. RNA samples that met the quality criteria were enriched for mRNA using oligo(dT) magnetic beads. For RNA sequencing library construction, the RNA was fragmented and reverse-transcribed into first-strand cDNA using random primers, followed by second-strand cDNA synthesis with dUTP incorporation. The double-stranded cDNA was subjected to end repair, A-tailing, adapter ligation, and polymerase chain reaction (PCR) amplification to generate the final library. The quality of the constructed library was verified with an Agilent 2100 Bioanalyzer and quantified via qPCR. Sequencing was then performed using the Illumina NovaSeq 6000 sequencer. The data is collected and stored in the Gene Expression Omnibus (GEO) database (Accession: GSE281894.).

### Statistical analysis

Each experiment was performed at least three times. Statistical analysis was performed using GraphPad Prism V.8. A two-tailed Student’s t-test or one-way analysis of variance was used to assess the mean differences between groups. A p-value < 0.05 was considered statistically significant.

## Supplementary information

Original data

Supplementary figure

Supplementary Material 1

Supplementary excel

## Data Availability

All data are available upon reasonable request.

## References

[1] Wang Y, Wang YP, Tay YC, Harris DC. Progressive adriamycin nephropathy in mice: sequence of histologic and immunohistochemical events. Kidney Int. 2000;58:1797–804. 11012915 10.1046/j.1523-1755.2000.00342.x

[2] D’agati VD, Kaskel FJ, Falk RJ. Focal segmental glomerulosclerosis. N Engl J Med. 2011;365:2398–411. 22187987 10.1056/NEJMra1106556

[3] Greka A, Mundel P. Cell biology and pathology of podocytes. Annu Rev Physiol. 2012;74:299–323. 22054238 10.1146/annurev-physiol-020911-153238 PMC3600372

[4] Zhou XJ, Rakheja D, Yu X, Saxena R, Vaziri ND, Silva FG. The aging kidney. Kidney Int. 2008;74:710–20. 18614996 10.1038/ki.2008.319

[5] Yu SM, He JC. Aged glomeruli: a link between PD-1 and podocytes. J Clin Invest. 2022;132:e162330. 10.1172/JCI162330 PMC9374372 35968780

[6] Pippin JW, Kaverina N, Wang Y, Eng DG, Zeng Y, Tran U, et al. Upregulated PD-1 signaling antagonizes glomerular health in aged kidneys and disease. J Clin Invest. 2022;132:e156250. 10.1172/JCI156250 PMC9374384 35968783

[7] Zhang L, Zhou F, Yu X, Zhu Y, Zhou Y, Liu J, et al. C/EBPα deficiency in podocytes aggravates podocyte senescence and kidney injury in aging mice. Cell Death Dis. 2019;10:684. 31527620 10.1038/s41419-019-1933-2 PMC6746733

[8] Born E, Lipskaia L, Breau M, Houssaini A, Beaulieu D, Marcos E, et al. Eliminating senescent cells can promote pulmonary hypertension development and progression. Circulation. 2023;147:650–66. 36515093 10.1161/CIRCULATIONAHA.122.058794

[9] Shi J, Yang C, Zhao K, Zhang J, Li P, Kong C, et al. NAT10 is involved in cardiac remodeling through ac4C-mediated transcriptomic regulation. Circ Res. 2023;133:989–1002. 37955115 10.1161/CIRCRESAHA.122.322244

[10] Zhang QR, Zhang JB, Shen F, Xue R, Yang RX, Ren TY, et al. Loss of NAT10 alleviates maternal high-fat diet-induced hepatic steatosis in male offspring of mice. Obesity. 2024;32:1349–61. 38816990 10.1002/oby.24041

[11] Bai Y, Zhang W, Hao L, Zhao Y, Tsai IC, Qi Y, et al. Acetyl-CoA-dependent ac(4)C acetylation promotes the osteogenic differentiation of LPS-stimulated BMSCs. Int Immunopharmacol. 2024;133:112124. 38663312 10.1016/j.intimp.2024.112124

[12] Qu Z, Pang X, Mei Z, Li Y, Zhang Y, Huang C, et al. The positive feedback loop of the NAT10/Mybbp1a/p53 axis promotes cardiomyocyte ferroptosis to exacerbate cardiac I/R injury. Redox Biol. 2024;72.103145. 38583415 10.1016/j.redox.2024.103145 PMC11002668

[13] Wang K, Zhou LY, Liu F, Lin L, Ju J, Tian PC, et al. PIWI-interacting RNA HAAPIR regulates cardiomyocyte death after myocardial infarction by promoting NAT10-mediated ac4C acetylation of Tfec mRNA. Adv Sci. 2022;9:2106058. 10.1002/advs.202106058 PMC8922123 35138696

[14] Balmus G, Larrieu D, Barros AC, Collins C, Abrudan M, Demir M, et al. Targeting of NAT10 enhances healthspan in a mouse model of human accelerated aging syndrome. Nat Commun. 2018;9:1–14. 10.1038/s41467-018-03770-3 PMC5923383 29703891

[15] Zhang ZQ, Zhang YW, Cai YJ, Li D, He JL, Feng ZH, et al. NAT10 regulates the LPS-induced inflammatory response via the NOX2-ROS-NF-κB pathway in macrophages. Biochim Biophys Acta Mol Basis Dis. 2023;1870:119521. 10.1016/j.bbamcr.2023.119521 37307924

[16] Kouzarides T, Viré E, Robson S, Breusegem SY, Kouzarides T, Jackson SP. Inhibition of the acetyltransferase NAT10 normalizes progeric and aging cells by rebalancing the Transportin-1 nuclear import pathway. Sci Signal. 2018;11:eaar5401. 10.1126/scisignal.aar5401 PMC6331045 29970603

[17] Cao Y, Yao M, Wu Y, Ma N, Liu H, Zhang B. N-acetyltransferase 10 promotes micronuclei formation to activate the senescence-associated secretory phenotype machinery in colorectal cancer cells. Transl Oncol. 2020;13.100783. 32428852 10.1016/j.tranon.2020.100783 PMC7232111

[18] Wang M, Zhang J, Qiu J, Ma X, Xu C, Wu Q, et al. Doxycycline decelerates aging in progeria mice. Aging Cell. 2024;23:e14188. 10.1111/acel.14188 PMC11258430 38686927

[19] Zhao CC, Sun X, Chen J, Geng BD. NAT10-mediated mRNA N4-acetylcytidine modification of MDR1 and BCRP promotes breast cancer progression. Thorac Cancer. 2024;15:820–9. 38409918 10.1111/1759-7714.15262 PMC10995701

[20] Wang XX, Zhao YM, Zhang QY, Zhao JX, Yin DH, Zhang ZZ, et al. Acetylcytidine modification of Amotl1 by N-acetyltransferase 10 contributes to cardiac fibrotic expansion in mice after myocardial infarction. Acta Pharm Sin. 2024;45:1425–37. 10.1038/s41401-024-01306-8 PMC11192918 38839936

[21] Hari P, Millar FR, Tarrats N, Birch J, Quintanilla A, Rink CJ, et al. The innate immune sensor Toll-like receptor 2 controls the senescence-associated secretory phenotype. Sci Adv. 2019;5.eaaw0254. 31183403 10.1126/sciadv.aaw0254 PMC6551188

[22] Lin H, Yan J, Wang Z, Hua F, Yu J, Sun W, et al. Loss of immunity-supported senescence enhances susceptibility to hepatocellular carcinogenesis and progression in Toll-like receptor 2-deficient mice. Hepatology. 2013;57:171–82. 22859216 10.1002/hep.25991

[23] Wang L, Chen R, Li G, Wang Z, Liu J, Liang Y, et al. FBW7 mediates senescence and pulmonary fibrosis through telomere uncapping. Cell Metab. 2020;32:860–77.e9. 33086033 10.1016/j.cmet.2020.10.004

[24] Fang Y, Chen B, Liu Z, Gong AY, Gunning WT, Ge Y, et al. Age-related GSK3β overexpression drives podocyte senescence and glomerular aging. J Clin Invest. 2022;132:1–16. 10.1172/JCI141848 PMC8843754 35166234

[25] Hasegawa Y, Saito T, Ogihara T, Ishigaki Y, Yamada T, Imai J, et al. Blockade of the nuclear factor-κB pathway in the endothelium prevents insulin resistance and prolongs life spans. Circulation. 2012;125:1122–33. 22302838 10.1161/CIRCULATIONAHA.111.054346

[26] Zoncu R, Efeyan A, Sabatini DM. mTOR: from growth signal integration to cancer, diabetes and ageing. Nat Rev Mol Cell Biol. 2011;12:21–35. 21157483 10.1038/nrm3025 PMC3390257

[27] Fang Y, Chen B, Gong AY, Malhotra DK, Gupta R, Dworkin LD, et al. The ketone body β-hydroxybutyrate mitigates the senescence response of glomerular podocytes to diabetic insults. Kidney Int. 2021;100:1037–53. 34246657 10.1016/j.kint.2021.06.031 PMC8889914

[28] Chen M, Fang Y, Ge Y, Qiu S, Dworkin L, Gong R. The redox-sensitive GSK3β is a key regulator of glomerular podocyte injury in type 2 diabetic kidney disease. Redox Biol. 2024;72.103127. 38527400 10.1016/j.redox.2024.103127 PMC10979123

[29] Zhang Y, Zhang N, Zou Y, Song C, Cao K, Wu B, et al. Deacetylation of Septin4 by SIRT2 (Silent Mating Type Information Regulation 2 Homolog-2) mitigates damaging of hypertensive nephropathy. Circ Res. 2023;132:601–24. 36786216 10.1161/CIRCRESAHA.122.321591 PMC9977266

[30] López-Otín C, Blasco MA, Partridge L, Serrano M, Kroemer G. Hallmarks of aging: an expanding universe. Cell. 2023;186:243–78. 36599349 10.1016/j.cell.2022.11.001

[31] Xie RH, Cheng L, Huang M, Huang L, Chen ZY, Zhang Q, et al. NAT10 drives cisplatin chemoresistance by enhancing ac4C-associated DNA repair in bladder cancer. Cancer Res. 2023;83:1666–83. 36939377 10.1158/0008-5472.CAN-22-2233

[32] Larrieu D, Britton S, Demir M, Rodriguez R, Jackson SP. Chemical inhibition of NAT10 corrects defects of laminopathic cells. Science. 2014;344:527–32. 24786082 10.1126/science.1252651 PMC4246063

[33] Zhang L, Li DQ. MORC2 regulates DNA damage response through a PARP1-dependent pathway. Nucleic Acids Res. 2019;47:8502–20. 31616951 10.1093/nar/gkz545 PMC6895267

[34] Liu HY, Liu YY, Yang F, Zhang L, Zhang FL, Hu X, et al. Acetylation of MORC2 by NAT10 regulates cell-cycle checkpoint control and resistance to DNA-damaging chemotherapy and radiotherapy in breast cancer. Nucleic Acids Res. 2020;48:3638–56. 32112098 10.1093/nar/gkaa130 PMC7144926

[35] Stucki M, Clapperton JA, Mohammad D, Yaffe MB, Smerdon SJ, Jackson SP. MDC1 directly binds phosphorylated histone H2AX to regulate cellular responses to DNA double-strand breaks. Cell. 2005;123:1213–26. 16377563 10.1016/j.cell.2005.09.038

[36] Singh I, Ozturk N, Cordero J, Mehta A, Hasan D, Cosentino C, et al. High mobility group protein-mediated transcription requires DNA damage marker γ-H2AX. Cell Res. 2015;25:837–50. 26045162 10.1038/cr.2015.67 PMC4493276

[37] Shrimp JH, Jing Y, Gamage ST, Nelson KM, Han J, Bryson KM, et al. Remodelin is a cryptic assay interference chemotype that does not inhibit NAT10-dependent cytidine acetylation. ACS Med Chem Lett. 2021;12:887–92. 34141066 10.1021/acsmedchemlett.0c00193 PMC8201477

[38] Wang G, Zhang M, Zhang Y, Xie Y, Zou J, Zhong J, et al. NAT10-mediated mRNA N4-acetylcytidine modification promotes bladder cancer progression. Clin Transl Med. 2022;12:e738. 35522942 10.1002/ctm2.738 PMC9076013

[39] Miao D, Shi J, Lv Q, Tan D, Zhao C, Xiong Z, et al. NAT10-mediated ac(4)C-modified ANKZF1 promotes tumor progression and lymphangiogenesis in clear-cell renal cell carcinoma by attenuating YWHAE-driven cytoplasmic retention of YAP1. Cancer Commun. 2024;44:361–83. 10.1002/cac2.12523 PMC10962679 38407929

[40] Wei W, Zhang SS, Han H, Wang XC, Zheng SY, Wang ZY, et al. NAT10-mediated ac4C tRNA modification promotes EGFR mRNA translation and resistance in cancer. Cell Rep. 2023;42:112810. 10.1016/j.celrep.2023.112810 37463108

[41] Pan Z, Bao Y, Hu M, Zhu Y, Tan C, Fan L, et al. Role of NAT10-mediated ac4C-modified HSP90AA1 RNA acetylation in ER stress-mediated metastasis and lenvatinib resistance in hepatocellular carcinoma. Cell Death Discov. 2023;9:56. 36765042 10.1038/s41420-023-01355-8 PMC9918514

[42] Liu R, Wubulikasimu Z, Cai R, Meng F, Cui Q, Zhou Y, et al. NAT10-mediated N4-acetylcytidine mRNA modification regulates self-renewal in human embryonic stem cells. Nucleic Acids Res. 2023;51:8514–31. 37497776 10.1093/nar/gkad628 PMC10484679

[43] Herrtwich L, Nanda I, Evangelou K, Nikolova T, Horn V, Sagar, et al. DNA damage signaling instructs polyploid macrophage fate in granulomas. Cell. 2016;167:1264–80.e18. 28084216 10.1016/j.cell.2016.09.054

