# Supplementary figures and images for: NAT10 mediates TLR2 to promote podocyte senescence in adriamycin-induced nephropathy
Source: Cell Death Dis. 2025 Mar 19;16(1):185. doi: 10.1038/s41419-025-07515-1 (PMC11923244; doi:10.1038/s41419-025-07515-1)

Figure 1.

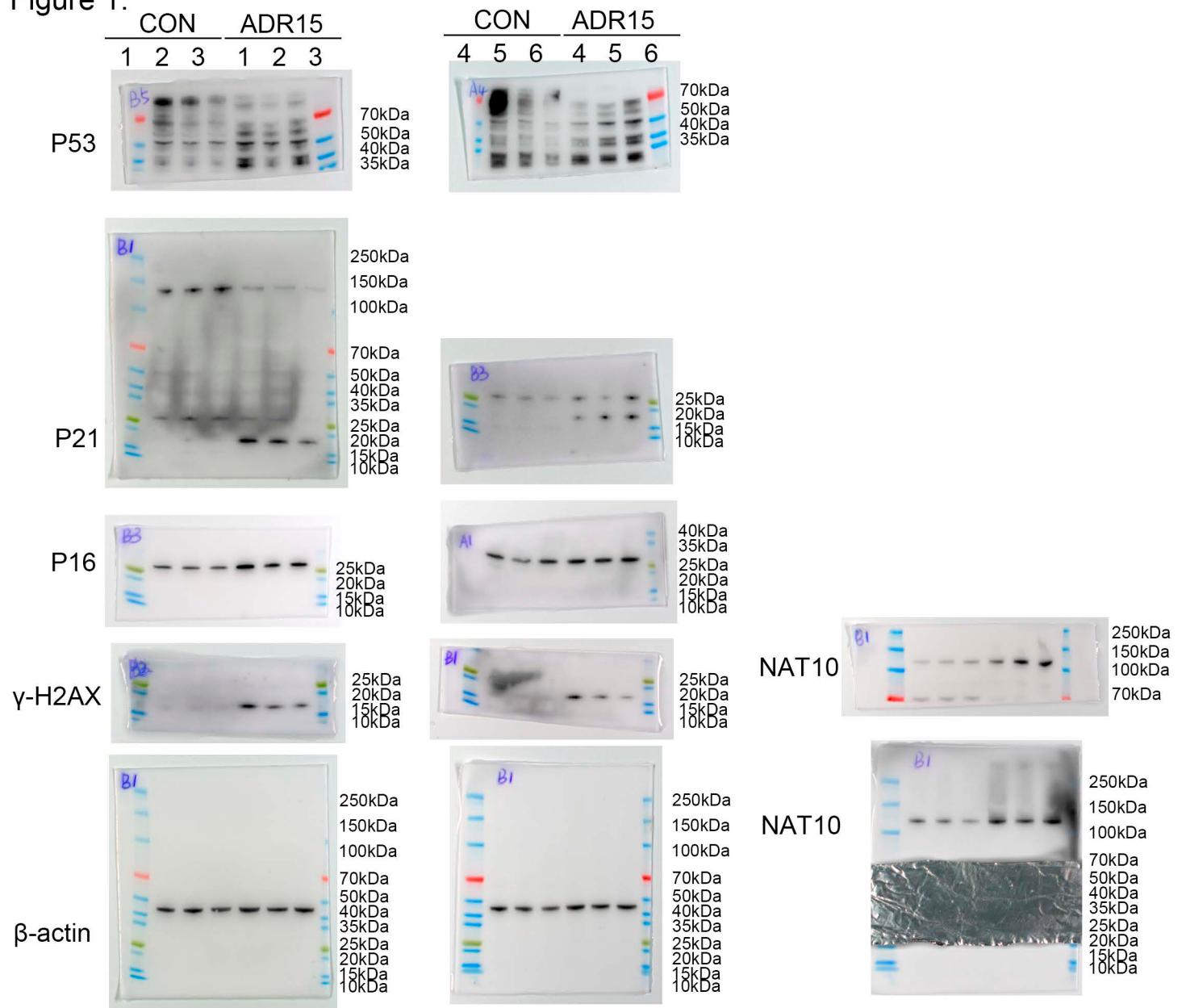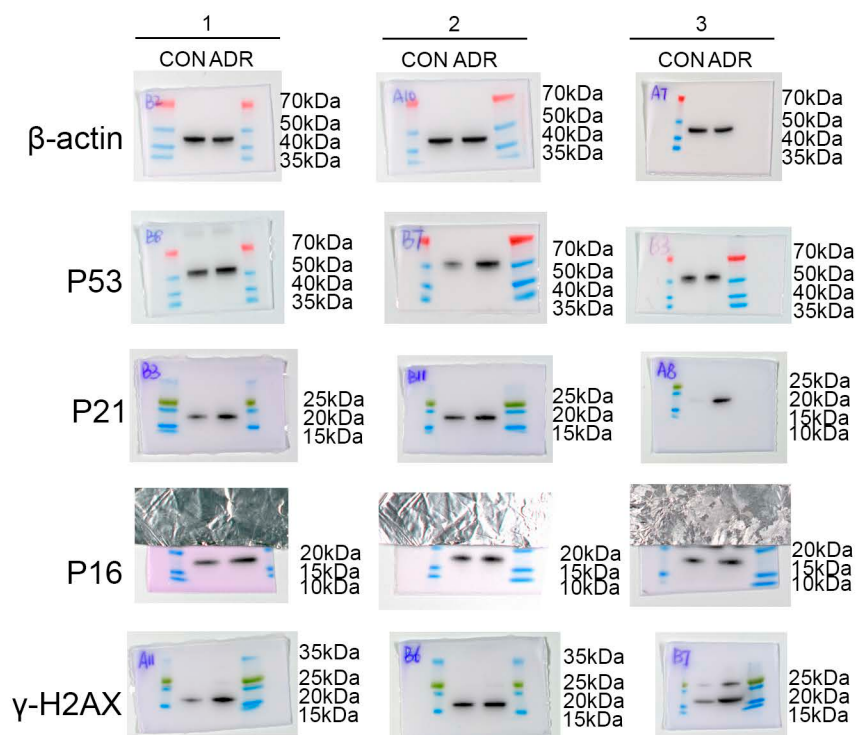

Figure 2.

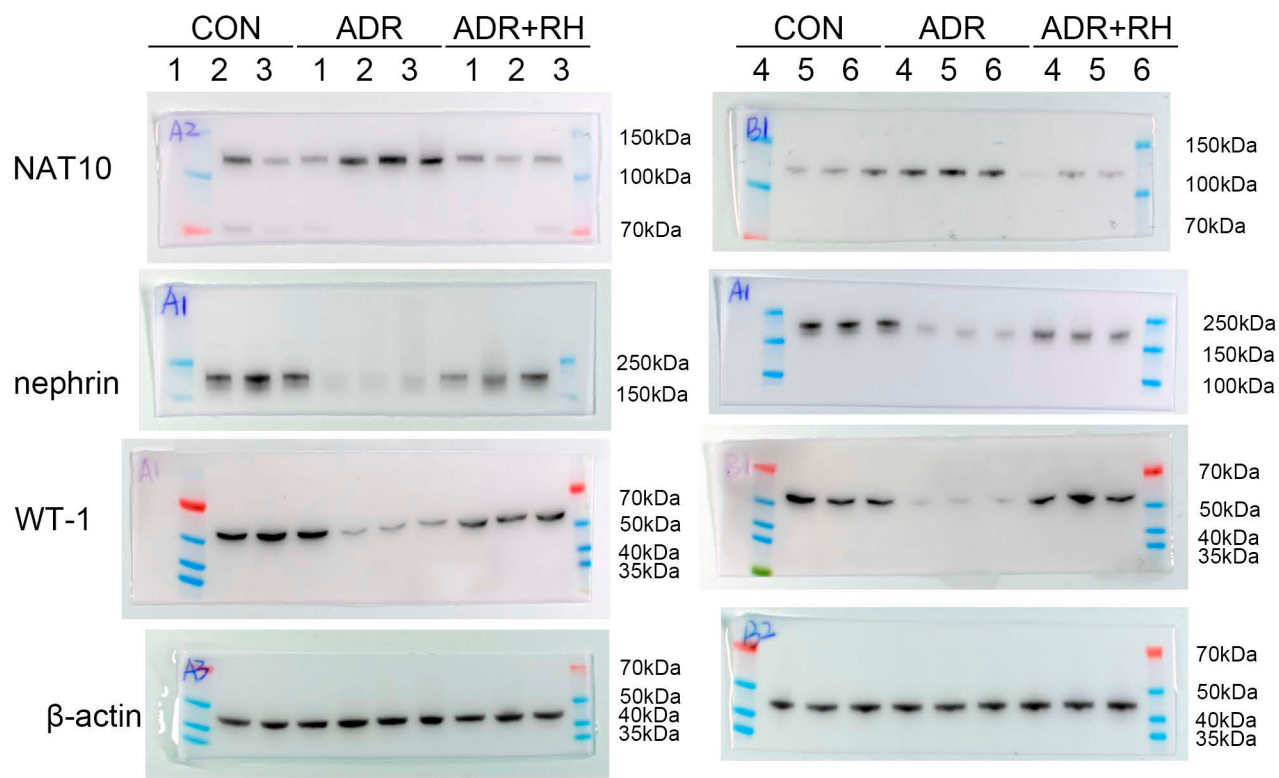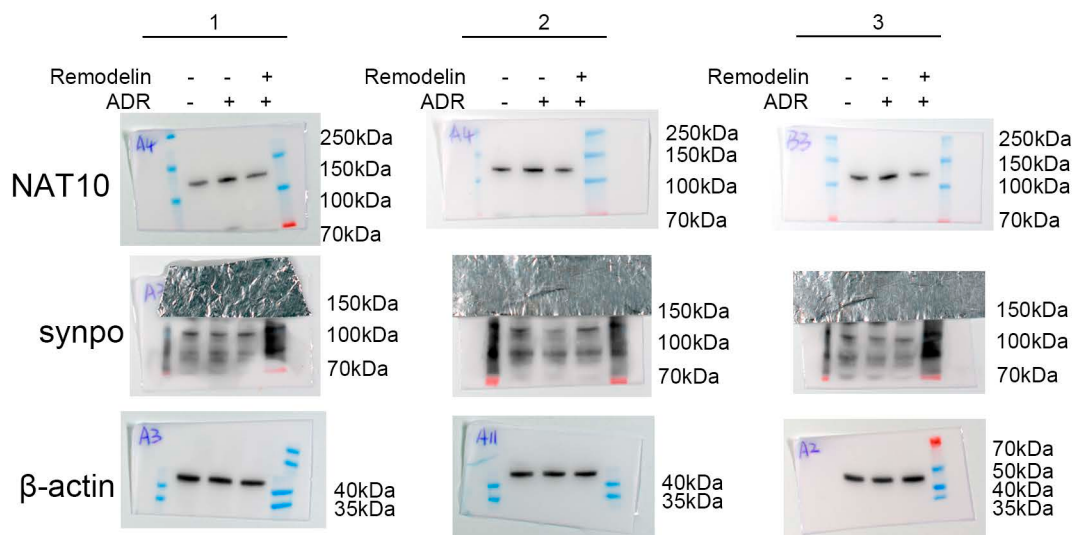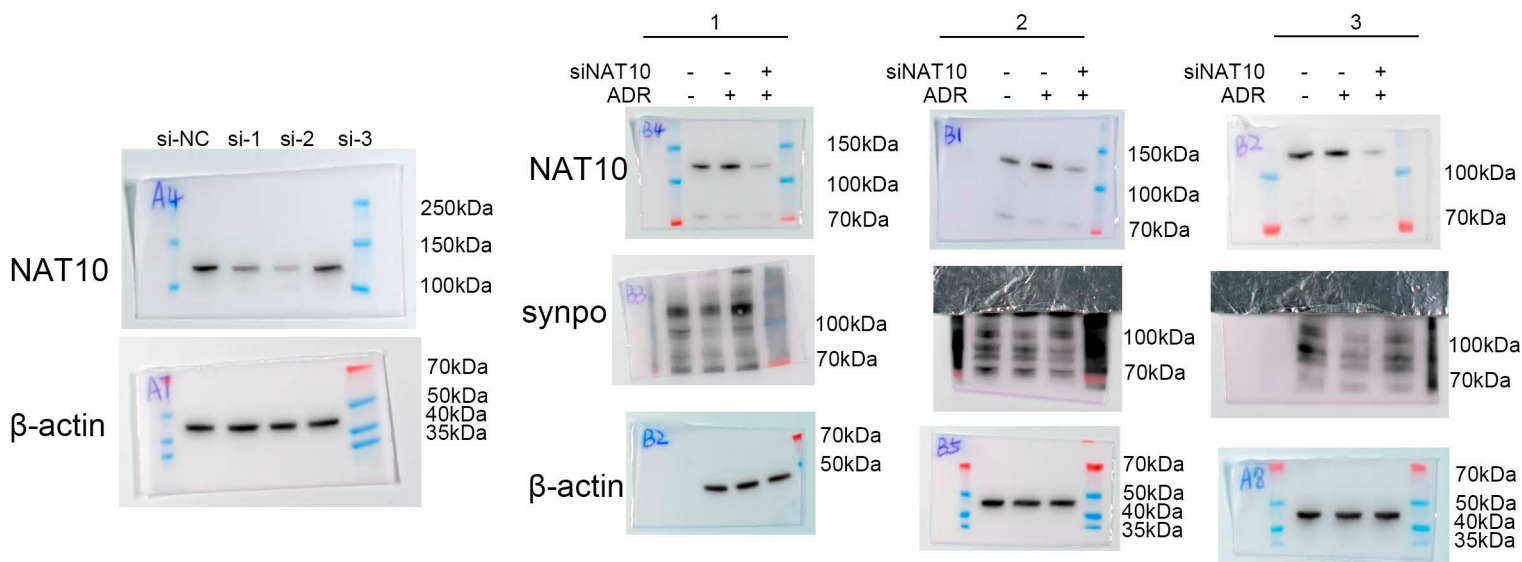

Figure 5.

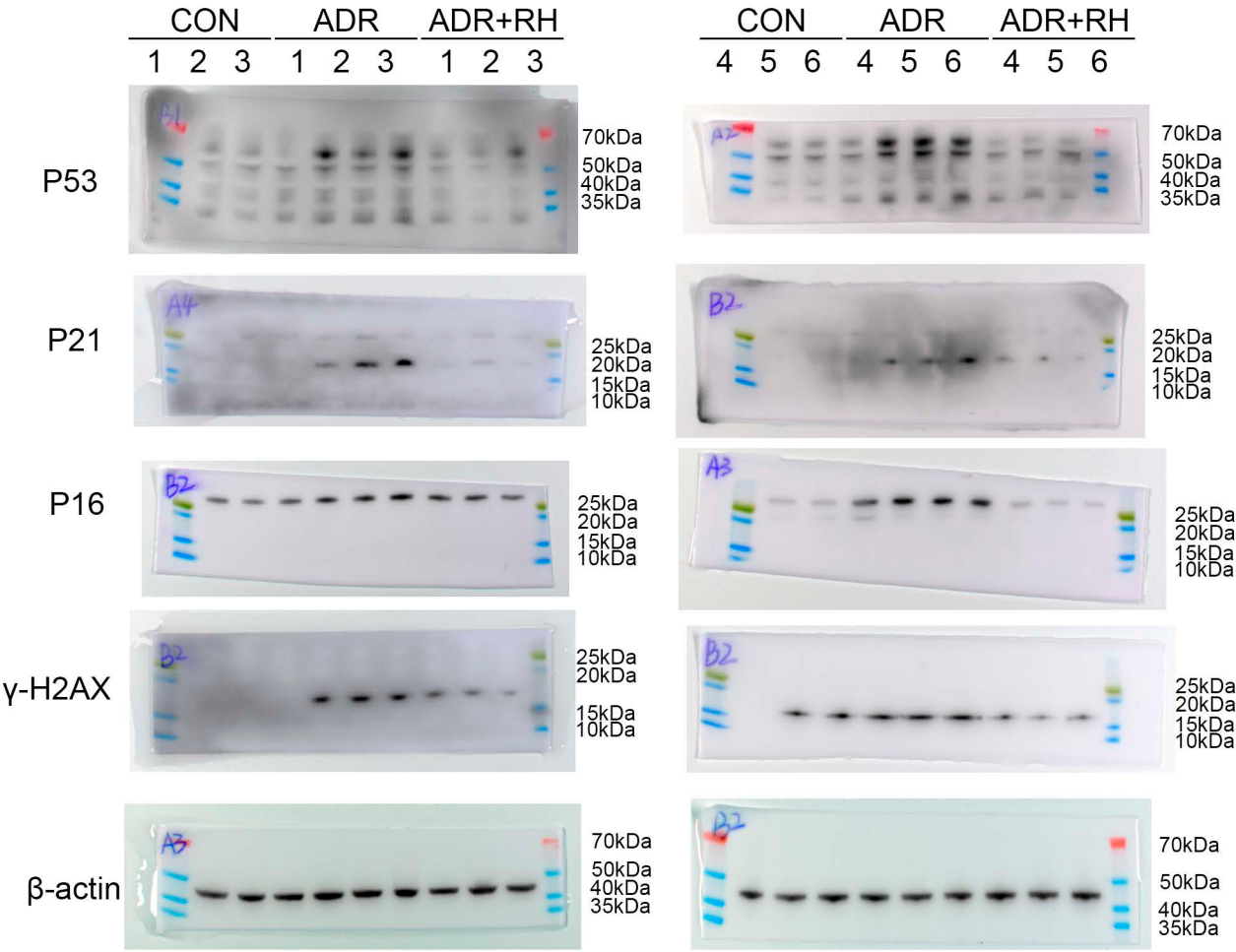

Figure 4.

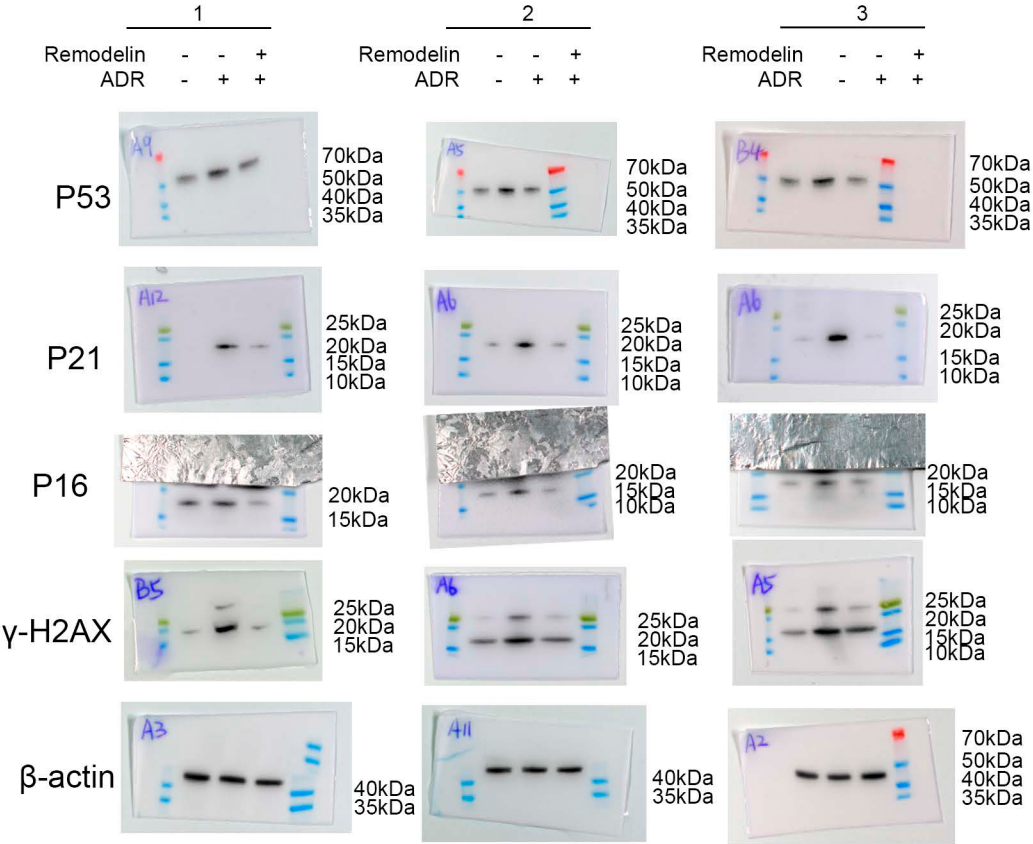

Figure 5.

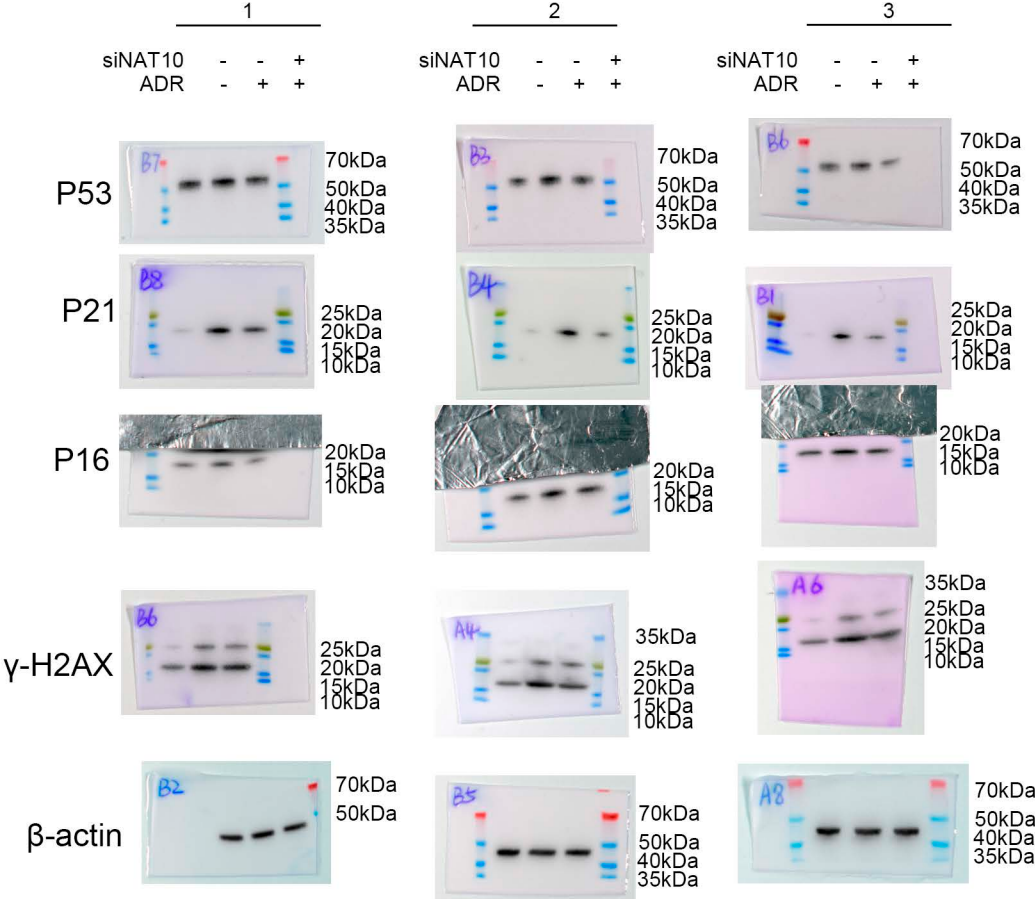

Figure 7.

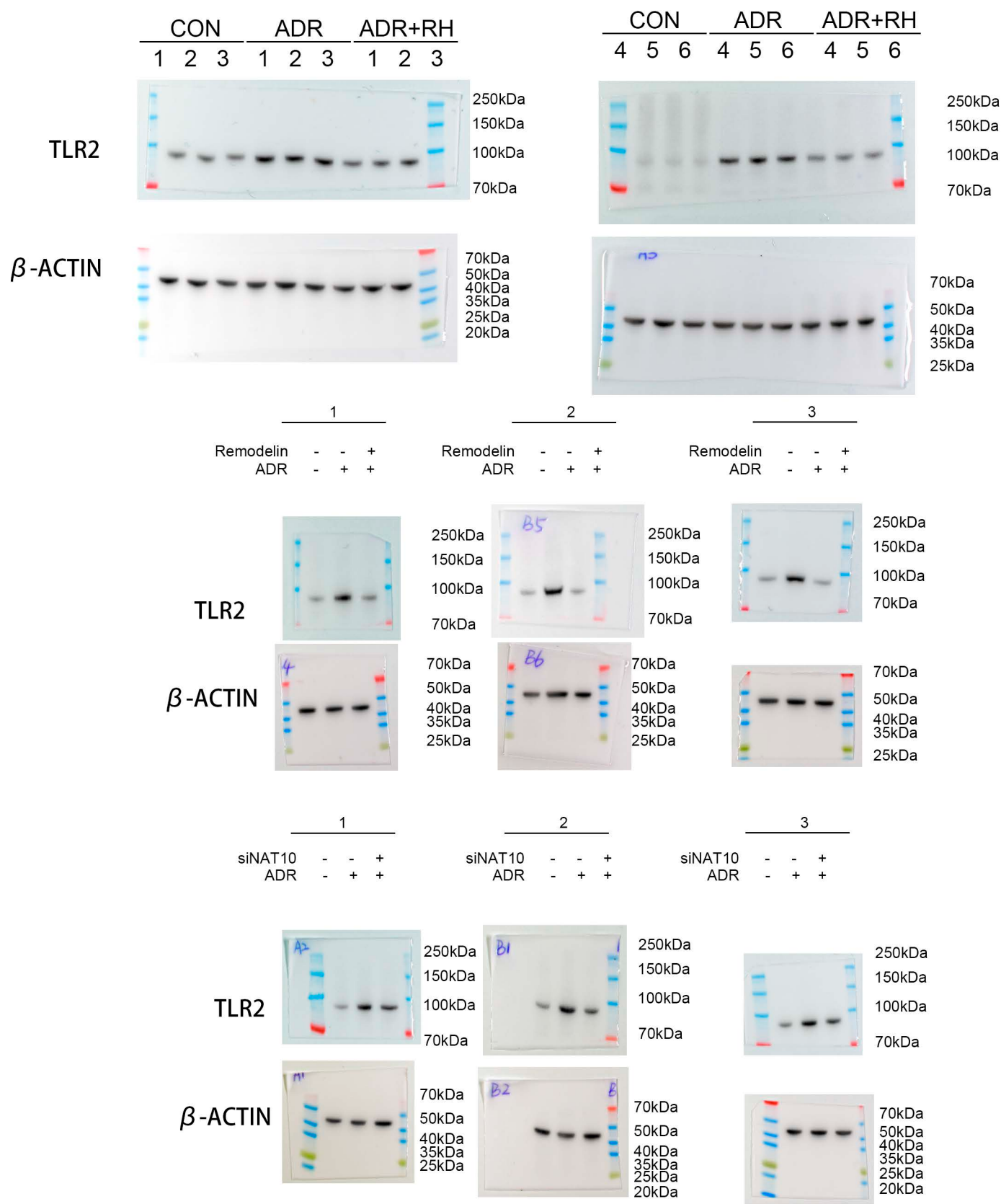

Figure 8.

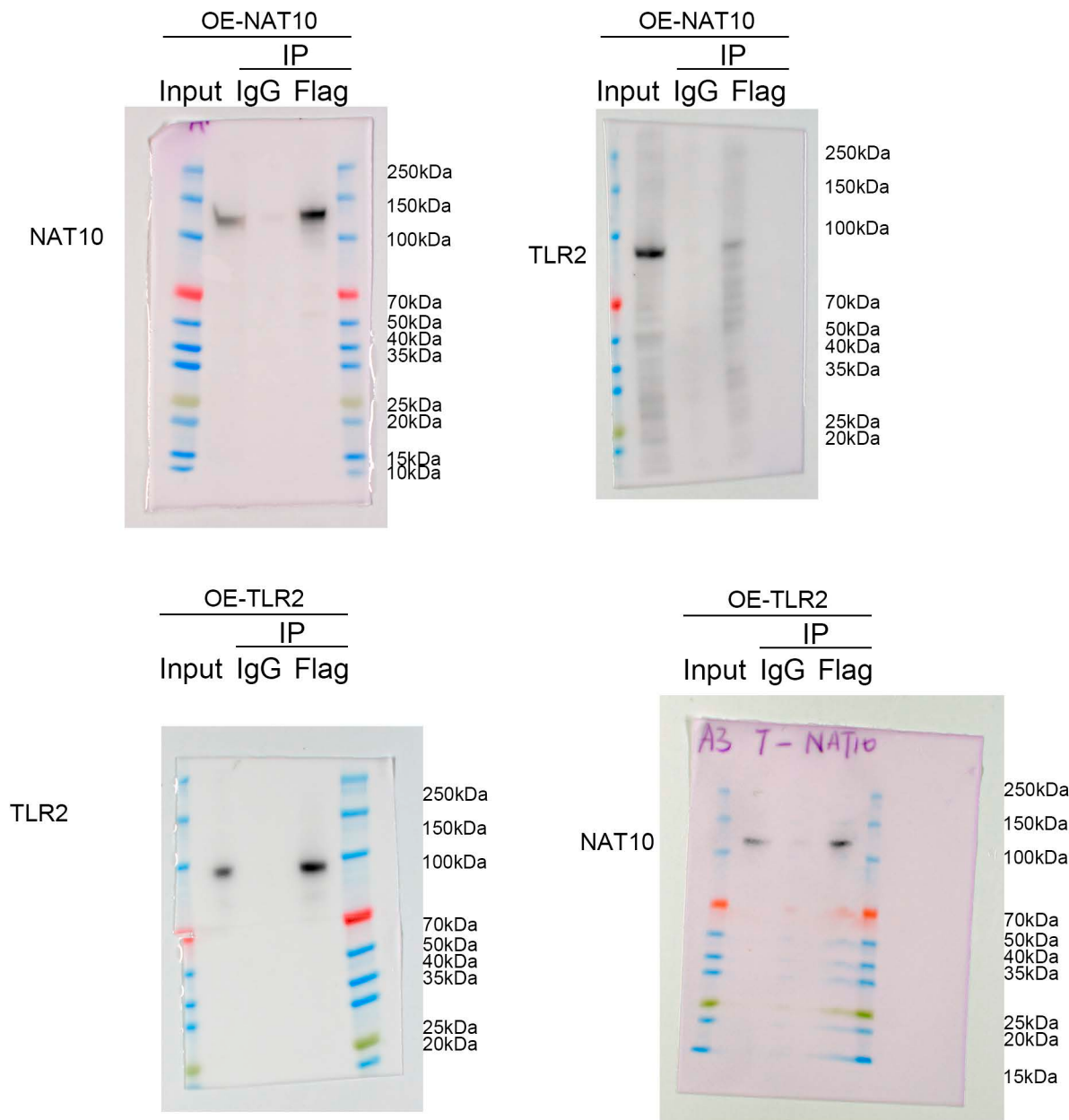

Figure 8.

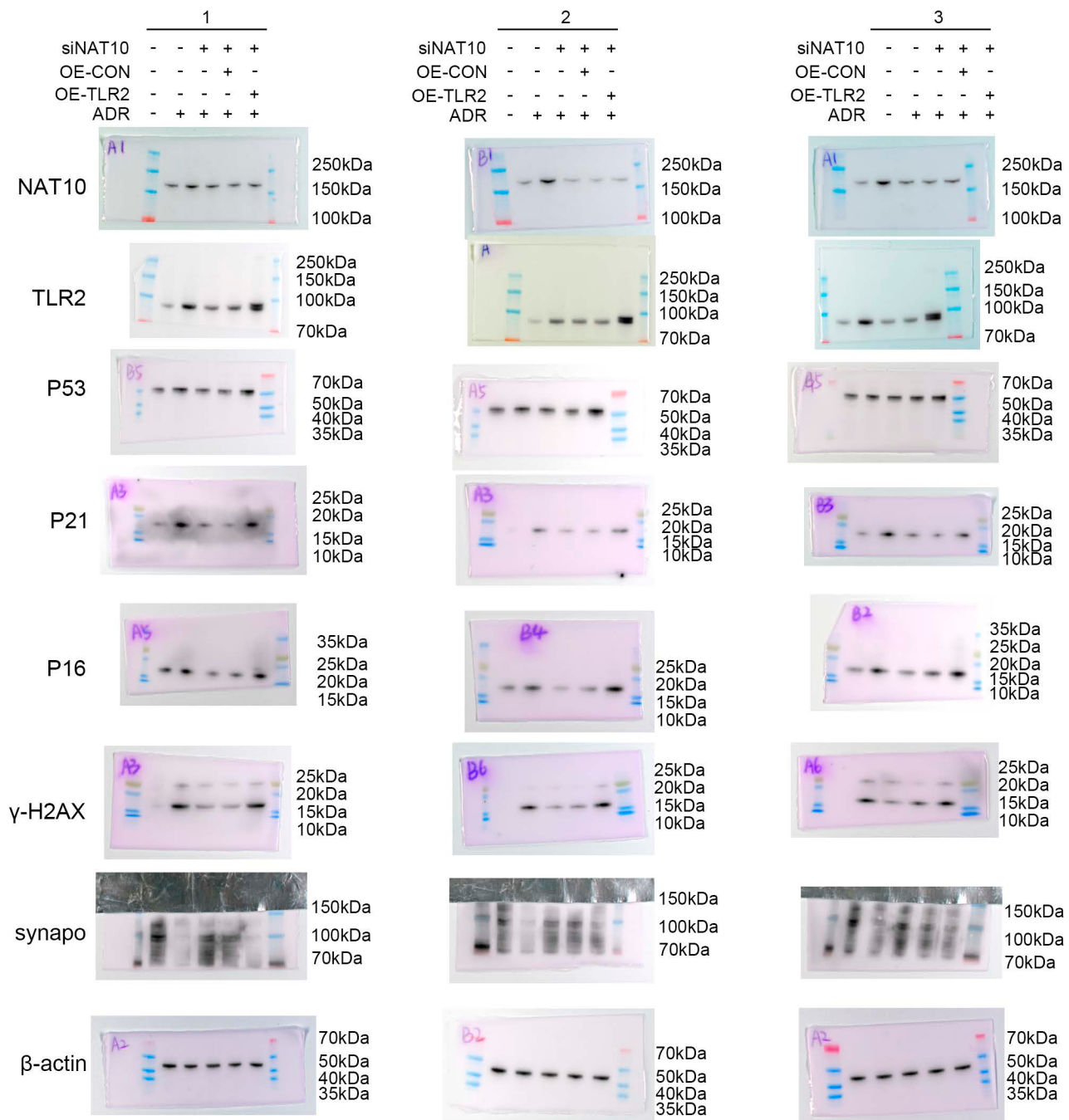

S-Figure 1.

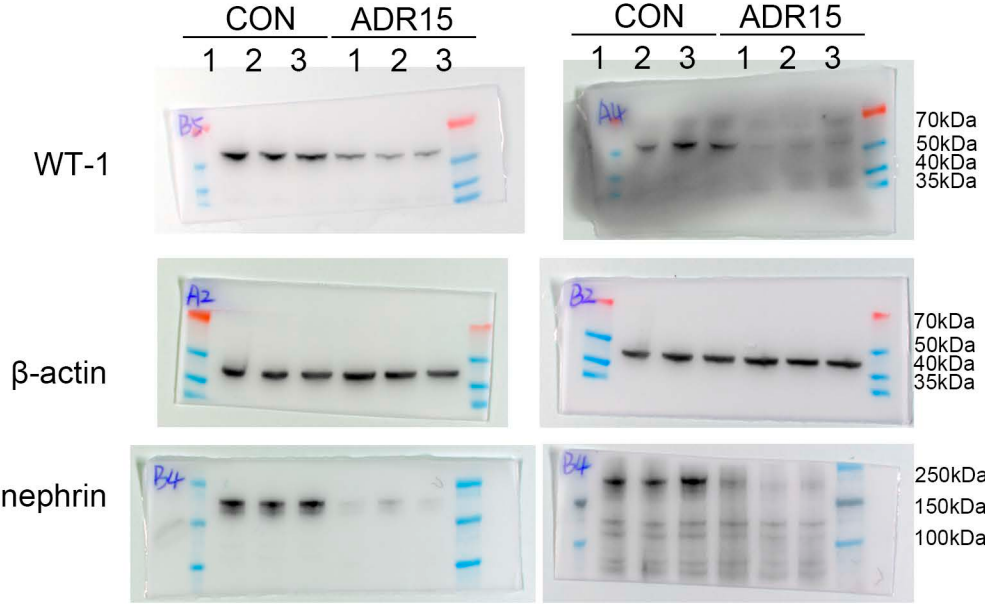

S-Figure 4.

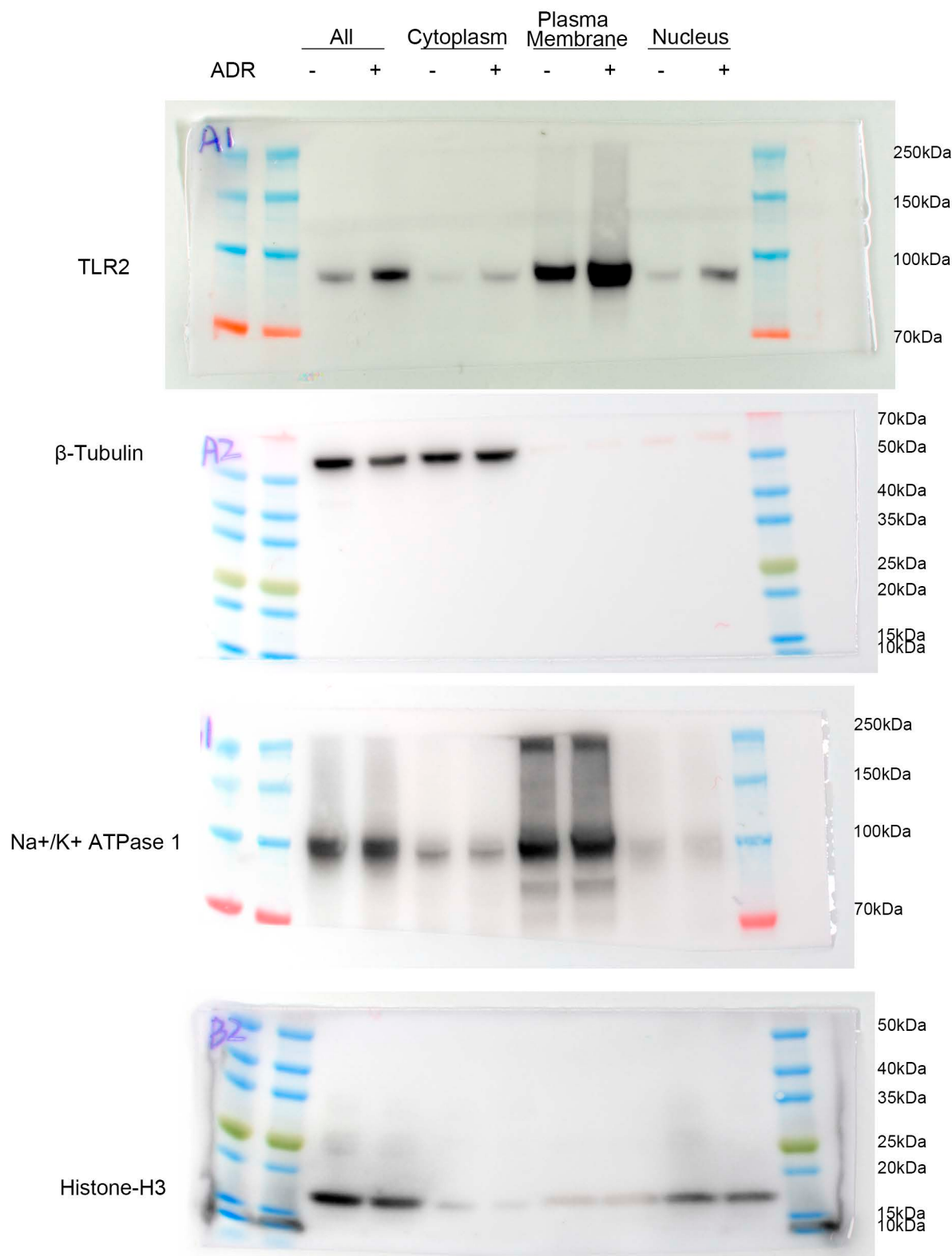

S-Figure 5

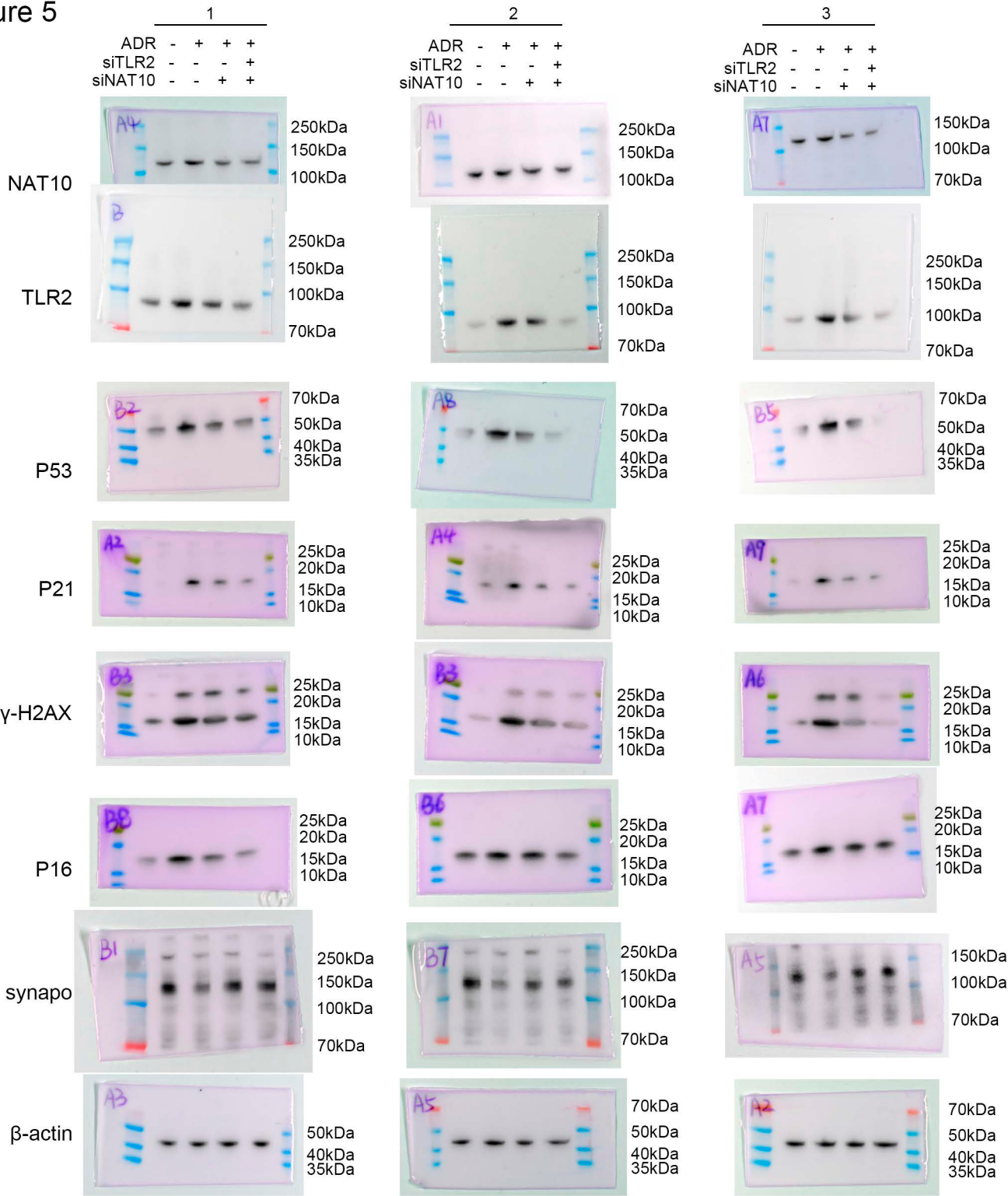

S-Figure 6.

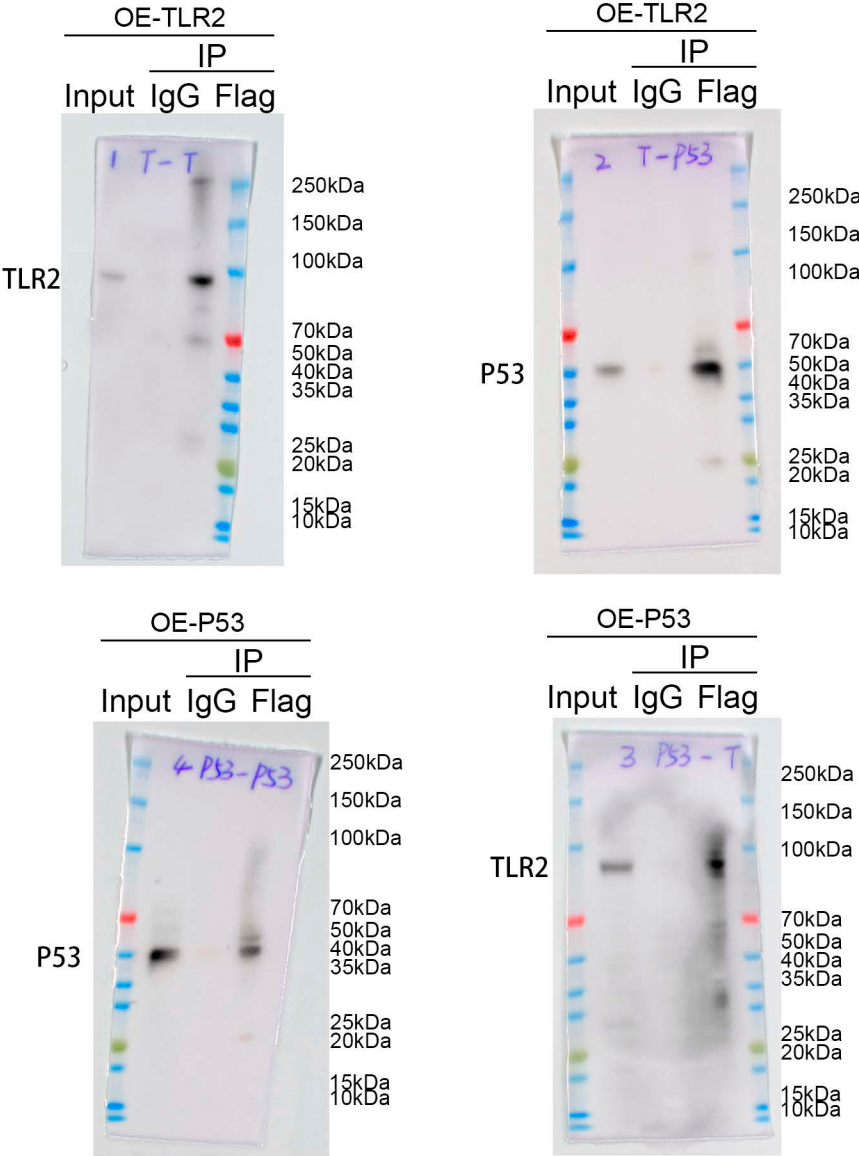

Supplement: Supplementary file 1 — Original data [file 41419_2025_7515_MOESM1_ESM.pdf]
