# Supplementary material for: NAT10 mediates TLR2 to promote podocyte senescence in adriamycin-induced nephropathy
Source: Cell Death Dis. 2025 Mar 19;16(1):185. doi: 10.1038/s41419-025-07515-1 (PMC11923244; doi:10.1038/s41419-025-07515-1)
Supplement: Supplementary file 2 — Supplementary figure [file 41419_2025_7515_MOESM2_ESM.pdf]

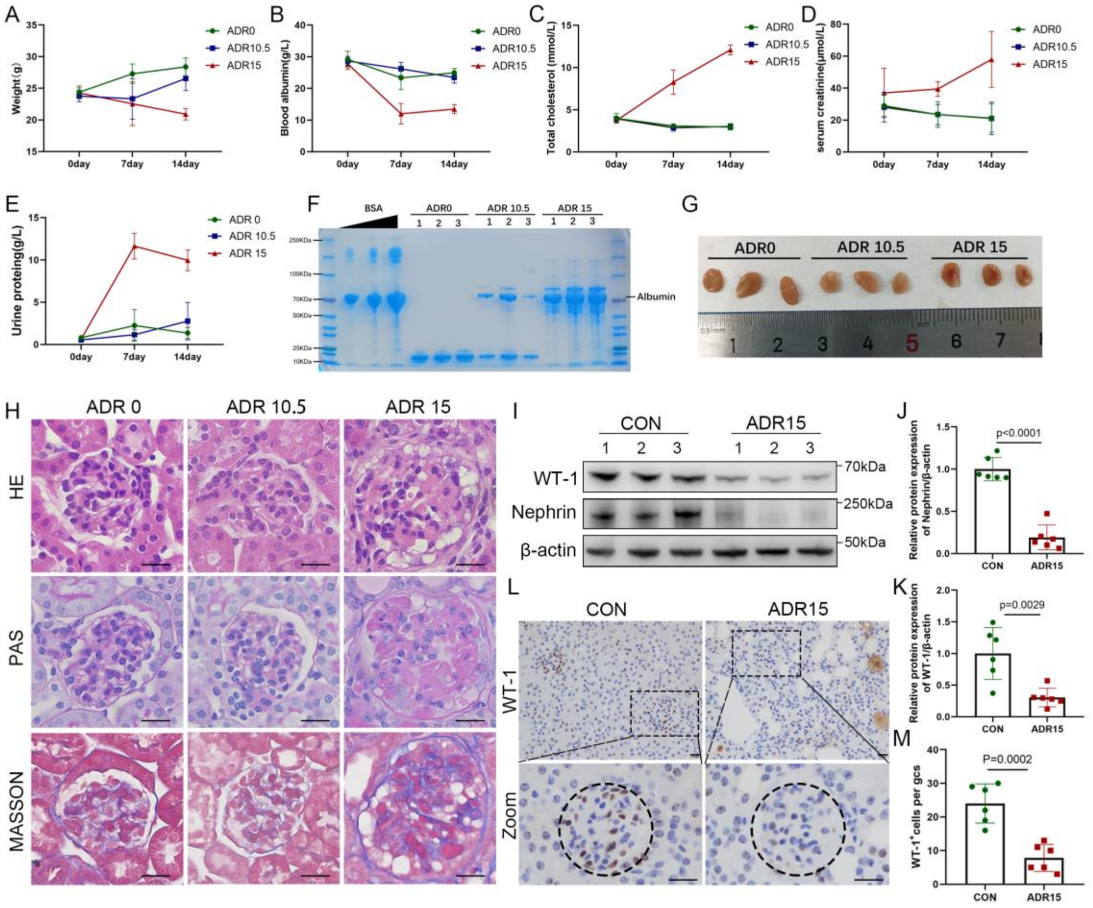

Supplementary Figure 1. Podocytopathy and proteinuria induced by adriamycin. (A) Body weight at various adriamycin concentrations and times. "ADR0" means the BALB/c mice were treated with an equal volume of saline solution. "ADR10.5" means the BALB/c mice were given Adriamycin at a concentration of 10.5mg/kg. "ADR15" means the BALB/c mice were injected with Adriamycin at a concentration of 15mg/kg. (B) Blood albumin in different groups. Green represents the CON group, blue represents the ADR10.5 group, and red represents the ADR15 group. (C) Total cholesterol in different groups. (D) Serum creatinine in different groups. (E) Urine protein in different groups. (F) Coomassie brilliant blue staining of urinary proteins in different groups. (G) Cross-section of kidney in different groups. (H) Mouse glomerulus staining included HE staining, Masson staining, and PAS staining. The scale bars = 20μm. (I) Western blot was used to detect WT-1 and nephrin expression in the CON group and ADR15 group (n=6). (J) Densitometric analysis of nephrin. The relative intensities of the bands were normalized to the intensities of the respective β-actin signal. (K) Densitometric analysis of WT-1. (L) Immunohistochemical detection of WT - 1 in the glomerulus and expression. The scale bars = 20μm. (M) The number of WT-1 positive cells was compared between the two groups.

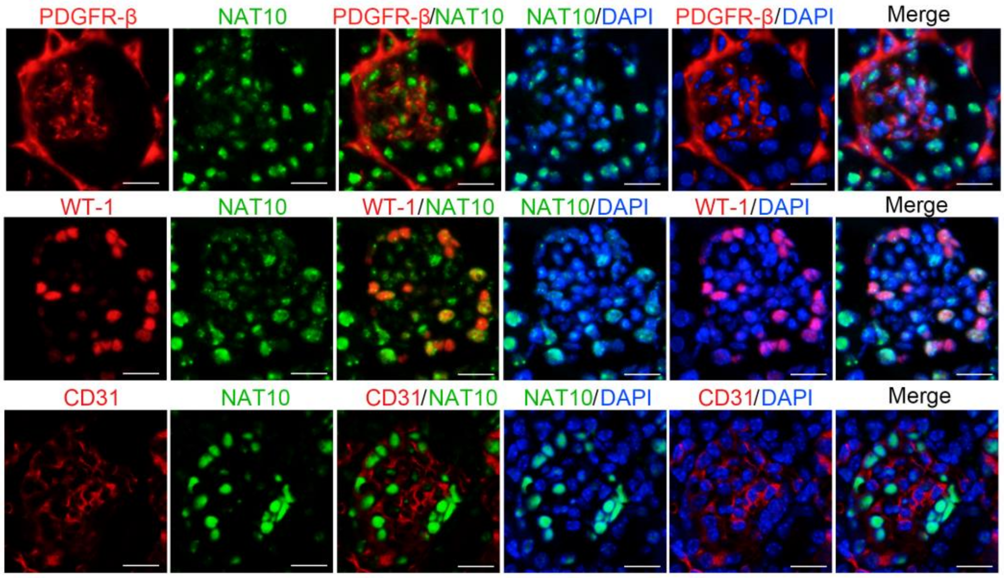

Supplementary Figure 2. Co-immunostaining of NAT10 with the marker proteins of various cells within the glomerulus. Green represents NAT10, red represents cell markers(PDGFR-β is a marker protein of mesangial cells, WT-1 is a marker protein of podocytes, and CD31 is a marker protein of endothelial cells), and Blue represents DAPI.The scale bars = 20μm.

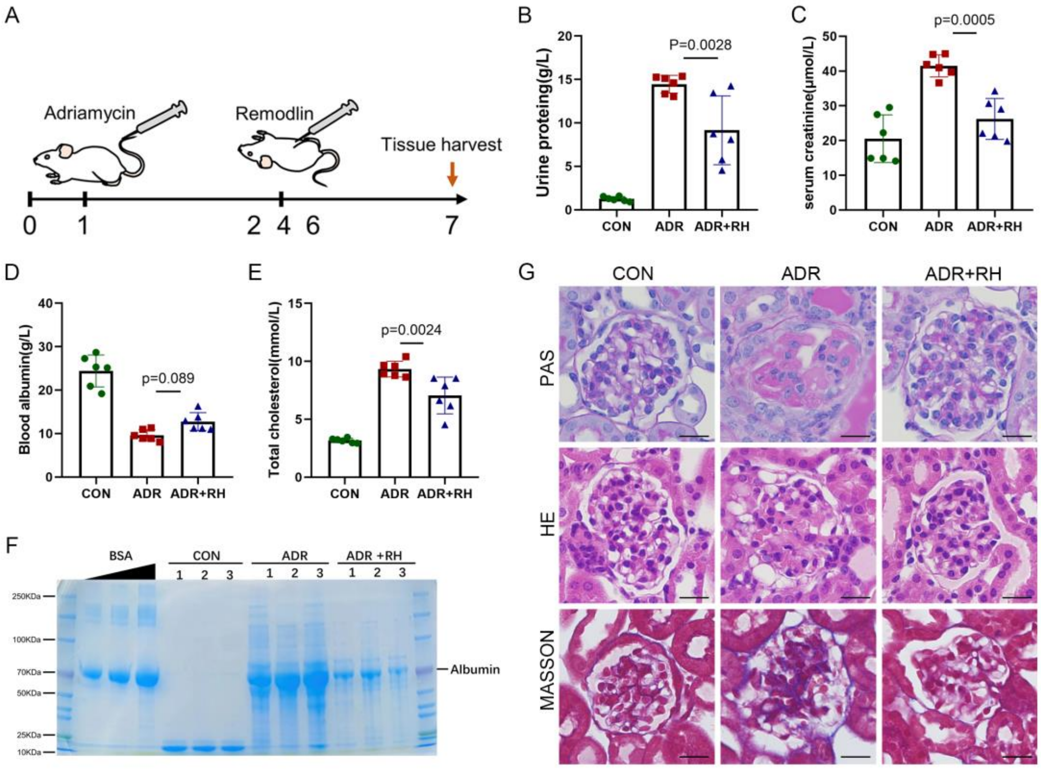

Supplementary Figure 3. Remodelin alleviates Adriamycin-induced kidney injury. (A) Experimental design. Adriamycin was injected intravenously on day 1. Stimulation concentration is 15mg/kg. Remodelin was injected intraperitoneally on days 2, 4, and day 6. Stimulation

concentration is 5mg/kg. Urine, blood, and kidney tissue were collected on day 7. (B) Urine protein in different groups on day 7. Green represents the control group (CON group), red represents the Adriamycin group (ADR group), and blue represents the Remodelin treatment group(ADR+RH group). (C) Serum creatinine in different gro ups. (D) Blood albumin in different groups. (E) Total cholesterol in different groups. (F) Coomassie brilliant blue staining of urinary proteins in different groups. (G) Representative micrographs show kidney injury at 7 days in different groups. The scale bars = 20μm.

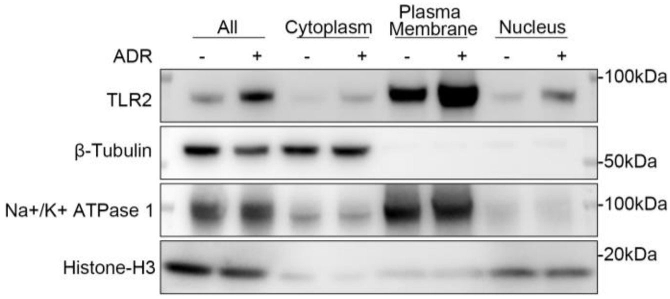

Supplementary Figure 4. Expression of TLR2 in podocytes induced by adriamycin. Western blot was used to detect TLR2, β-Tubulin, Na+/K+ ATPase 1 and Histone-H3 expression in different groups The stimulation condition of adriamycin was 0.5μg/ml/12h.

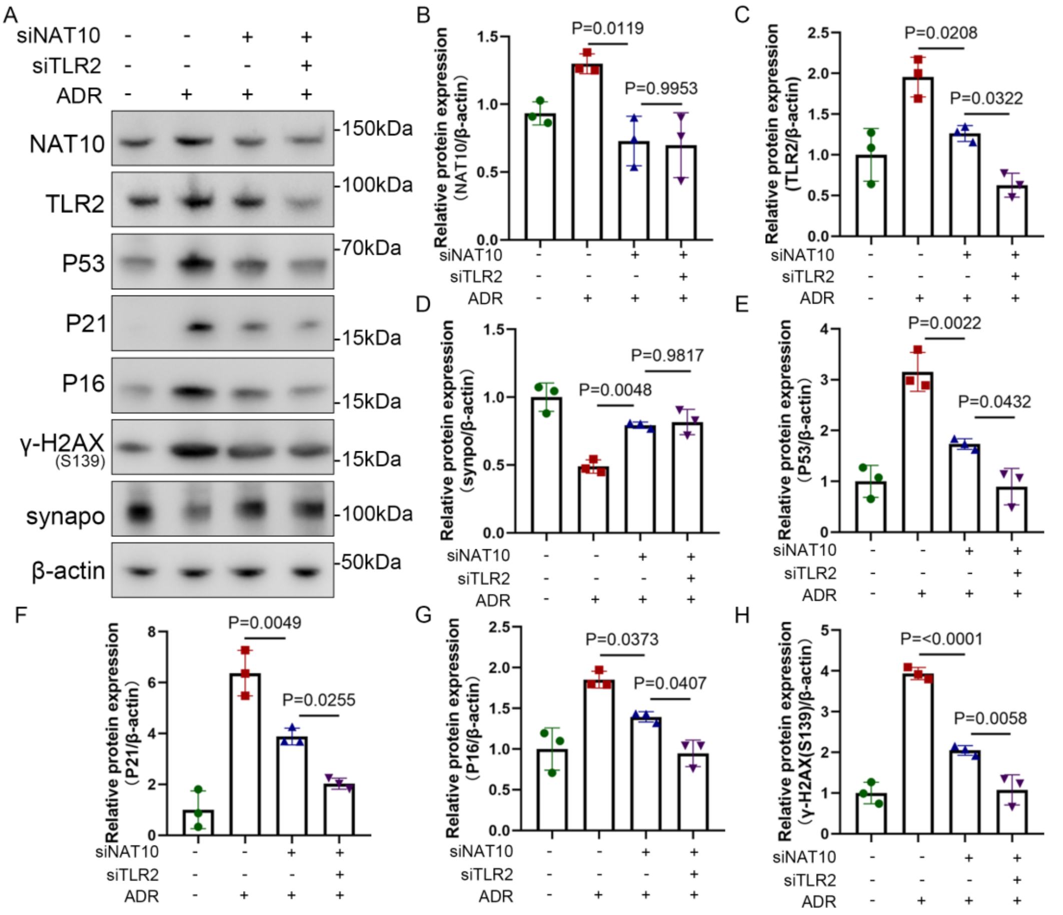

Supplementary Figure 5. Inhibition of TLR2 further enhanced the protective effect of NAT10 knockdown in podocytes upon adriamycin injury. (A) Western blot was used to detect NAT10, TLR2, P53, P21, P16, and γH2AX(S139) expression in different groups (n=3). The stimulation time of siNAT10 and siTLR2 was 36h. The stimulation condition of adriamycin was 0.5μg/ml/12h. (B) Densitometric analysis of NAT10. The relative intensities of the bands were normalized to the intensities of the respective β-actin signal. (C) Densitometric analysis of TLR2. (D) Densitometric analysis of Synaptopodin. (E) Densitometric analysis of P53. (F) Densitometric analysis of P21. (G) Densitometric analysis of P16. (H) Densitometric analysis of γH2AX(S139).

A

Interface residues	model_1	model_2	model_3	model_4	model_5	model_6	model_7	model_8	model_9	model_10
Docking Score	-309.23	-307.76	-305.45	-293.16	-292.68	-280.82	-275.43	-273.51	-269.65	-265
Confidence Score	0.9603	0.9591	0.9573	0.946	0.9455	0.9319	0.9247	0.922	0.9163	0.9089
Ligand rmsd (Å)	53.36	35.13	39.74	54.01	54.04	55.32	47.93	40.73	44.41	40.46

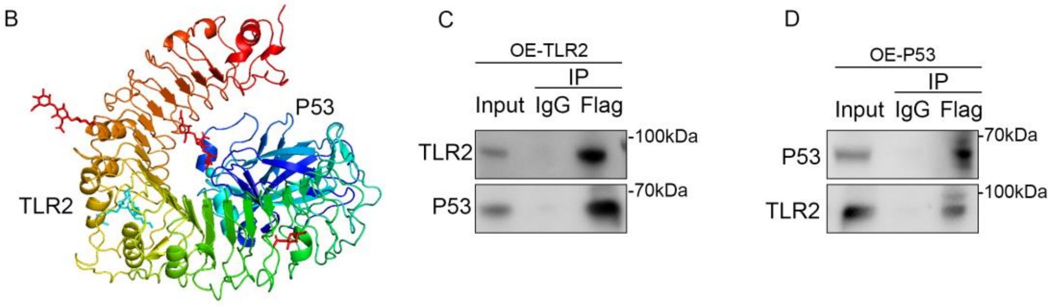

Supplementary Figure 6. Direct interaction between TLR2 and NAT10. (A) Information on the top ten docking models ranked by docking scores. The docking scores are calculated by the iterative scoring function ITScorePP or ITScorePR. Confidence score = 1.0/[1.0+e0.02*(Docking_Score+150)]. The ligand RMSDs are calculated by comparing the ligands in the docking models with the input or modeled structures. (B) Molecular docking model of TLR2 and P53. (C) Immunoprecipitation after transfection with a flag-tagged plasmid overexpressing TLR2. TLR2 and P53 were detected by WB. (D) Immunoprecipitation after transfection with a flag-tagged plasmid overexpressing P53. P53 and TLR2 were detected by WB.
