# Supplementary material for: NAT10 mediates TLR2 to promote podocyte senescence in adriamycin-induced nephropathy
Source: Cell Death Dis. 2025 Mar 19;16(1):185. doi: 10.1038/s41419-025-07515-1 (PMC11923244; doi:10.1038/s41419-025-07515-1)
Supplement: Supplementary file 3 — Supplementary Material 1 [file 41419_2025_7515_MOESM3_ESM.pdf]

>Tlr2-201 ENSMUSE00000590378 exon:protein_coding
CGGAGCCTCTGGACTTTCAGTTCTGTTTTGCCTGCCCTGTGGCTCCTGCCAGCTCTGATG
CCAGGCTCCGTTCCCCTTGCAGACCCCTGTGGGCGGCGCTTGCCGCACCGGGGGCGGTGC
TGGCGACCGGGAAGTTCGGGGCCCTGACCTGGGGACATCCCCTTCCCTCACTTCCAGGTC
TTCAGTCTTCCTAGGCTGGTGCCCAGATGGCTAGTGGGCACGGGGAGCGGCGGCTGGAGG
ACTCCTAG
>Tlr2-201 ENSMUSE00000508310 exon:protein_coding
GCTCCGGGCAGGCGGTCACTGGCAGGAGATGTGTCCGCAATCATAGTTTCTGATGGTGAA
GGTTGGACGGCAGTCTCTGCGACCTAGAAGTGGAAAAGATGTCGTTCAAGGAGGTGCGGA
CTGTTTCCTTCTGACCAGGATCTTGTTTCTGAGTGTAGGGGCTTCACTTCTCTGCTTTTC
GTTCATCTCTGGAGCATCCGAATTGCATCACCGGTCAGAAAACAACTTACCGAAACCTCA
GACAAAGC
>Tlr2-201 ENSMUSE00000441912 exon:protein_coding
GTCAAATCTCAGAGGATGCTACGAGCTCTTTGGCTCTTCTGGATCTTGGTGGCCATAACA
GTCCTCTTCAGCAAACGCTGTTCTGCTCAGGAGTCTCTGTCATGTGATGCTTCTGGGGTG
TGTGATGGCCGCTCCAGGTCTTTCACCTCTATTCCCTCCGGACTCACAGCAGCCATGAAA
AGCCTTGACCTGTCTTTCAACAAGATCACCTACATTGGCCATGGTGACCTCCGAGCGTGT
GCGAACCTCCAGGTTCTGATGTTGAAGTCCAGCAGAATCAATACAATAGAGGGAGACGCC
TTTTATTCTCTGGGCAGTCTTGAACATTTGGATTTGTCTGATAATCACCTATCTAGTTTA
TCTTCCTCCTGGTTCGGGCCCCTTTCCTCTTTGAAATACTTAAACTTAATGGGAAATCCT
TACCAGACACTGGGGGTAACATCGCTTTTTCCCAATCTCACAAATTTACAAACCCTCAGG
ATAGGAAATGTAGAGACTTTCAGTGAGATAAGGAGAATAGATTTTGCTGGGCTGACTTCT
CTCAATGAACTTGAAATTAAGGCATTAAGTCTCCGGAATTATCAGTCCCAAAGTCTAAAG
TCGATCCGCGACATCCATCACCTGACTCTTCACTTAAGCGAGTCTGCTTTCCTGCTGGAG
ATTTTTGCAGATATTCTGAGTTCTGTGAGATATTTAGAACTAAGAGATACTAACTTGGCC
AGGTTCCAGTTTTCACCACTGCCCGTAGATGAAGTCAGCTCACCGATGAAGAAGCTGGCA
TTCCGAGGCTCGGTTCTCACTGATGAAAGCTTTAACGAGCTCCTGAAGCTGTTGCGTTAC
ATCTTGGAACTGTCGGAGGTAGAGTTCGACGACTGTACCCTCAATGGGCTCGGCGATTTC
AACCCCTCGGAGTCAGACGTAGTGAGCGAGCTGGGTAAAGTAGAAACAGTCACTATCCGG
AGGTTGCATATCCCCCAGTTCTATTTGTTTTATGACCTGAGTACTGTCTATTCCCTCCTG
GAGAAGGTGAAGCGAATCACAGTAGAGAACAGCAAGGTCTTCCTGGTTCCCTGCTCGTTC
TCCCAGCATTTAAAATCATTAGAATTCTTAGACCTCAGCGAAAATCTGATGGTTGAAGAA
TATTTGAAGAACTCAGCCTGTAAGGGAGCCTGGCCTTCTCTACAAACCTTAGTTTTGAGC
CAGAATCATTTGAGATCAATGCAAAAAACAGGAGAGATTTTGCTGACTCTGAAAAACCTG
ACCTCTCTTGACATCAGCAGGAACACTTTTCATCCGATGCCCGACAGCTGTCAGTGGCCA
GAAAAGATGCGCTTCCTGAATTTGTCCAGTACAGGGATCCGGGTGGTAAAAACGTGCATT
CCTCAGACGCTGGAGGTGTTGGATGTTAGTAACAACAATCTTGACTCATTTTCTTTGTTC
TTGCCTCGGCTGCAAGAGCTCTATATTTCCAGAAATAAGCTGAAAACACTCCCAGATGCT
TCGTTGTTCCCTGTGTTGCTGGTCATGAAAATCAGAGAGAATGCAGTAAGTACTTTCTCT
AAAGACCAACTTGGTTCTTTTCCCAAACTGGAGACTCTGGAAGCAGGCGACAACCACTTT
GTTTGCTCCTGCGAACTCCTATCCTTTACTATGGAGACGCCAGCTCTGGCTCAAATCCTG
GTTGACTGGCCAGACAGCTACCTGTGTGACTCTCCGCCTCGCCTGCACGGCCACAGGCTT
CAGGATGCCCGGCCCTCCGTCTTGGAATGTCACCAGGCTGCACTGGTGTCTGGAGTCTGC
TGTGCCCTTCTCCTGTTGATCTTGCTCGTAGGTGCCCTGTGCCACCATTTCCACGGACTG

TGGTACCTGAGAATGATGTGGGCGTGGCTCCAGGCCAAGAGGAAGCCCAAGAAAGCTCCC
TGCAGGGACGTTTGCTATGATGCCTTTGTTTCCTACAGTGAGCAGGATTCCCATTGGGTG
GAGAACCTCATGGTCCAGCAGCTGGAGAACTCTGACCCGCCCTTTAAGCTGTGTCTCCAC
AAGCGGGACTTCGTTCCGGGCAAATGGATCATTGACAACATCATCGATTCCATCGAAAAG
AGCCACAAAACTGTGTTCGTGCTTTCTGAGAACTTCGTACGGAGCGAGTGGTGCAAGTAC
GAACTGGACTTCTCCCACTTCAGGCTCTTTGACGAGAACAACGACGCGGCCATCCTTGTT
TTGCTGGAGCCCATTGAGAGGAAAGCCATTCCCCAGCGCTTCTGCAAACTGCGCAAGATA
ATGAACACCAAGACCTACCTGGAGTGGCCCTTGGATGAAGGCCAGCAGGAAGTGTTTTGG
GTAAATCTGAGAACTGCAATAAAGTCCTAGGTTCTCCACCCAGTTCCTGACTTCCTTAAC
TAAGGTCTTTGTGACACAAACTGTAACAAAGTTTATAAGTAACATAGAATTGTATTATTG
AGGATATTAACTATGGGTTTTGTCTTGAATACTGTTATATAAATATGTGACATCAGGA

PACES Prediction of acetylation sites in mRNA

Result:

ID	Sequence	Start	End	Score thresholds: 0.4126
null	no acetylated region	null	null	null

Citation:Zhao W., Zhou Y., Cui Q. & Zhou Y. PACES: prediction of N4-acetylcytidine (ac4C) modification sites in mRNA. Sci Rep 9, 11112, doi:10.1038/s41598-019-47594-7 (2019).
